# Optimizing electrokinetic remediation for pollutant removal and electroosmosis/dewatering using lateral anode configurations

**DOI:** 10.1038/s41598-024-75060-6

**Published:** 2024-10-25

**Authors:** Ahmed Abou-Shady, Doaa Eissa, Osama Abd-Elmottaleb, Asmaa K. Bahgaat, Mohamed A. Osman

**Affiliations:** Soil Physics and Chemistry Department, Water Resources and Desert Soils Division, Desert Research Center, El-Matariya, Cairo, 4540031 Egypt

**Keywords:** Soil electrokinetics, The PCPSS, Process intensification, Process optimization, Inorganic pollutants, Electroosmosis/dewatering, Environmental sciences, Chemistry, Engineering

## Abstract

Soil electrokinetics (SEK) research has been widely used in various fields such as soil remediation, dewatering, land restoration, geophysics, sedimentation, pollution prevention, consolidation, and seed germination. According to our most recent published research on SEK process design modifications during the last 30 years (1993–2022), more than 150 designs have been introduced to assure SEK’s maximum performance. Incorporating lateral electrodes/anodes was not documented in the existing literature, which motivated us to investigate the output of this design. In this study, we aimed to enhance the performance of the perforated cathode pipe soil electrokinetic remediation (SEKR) system (PCPSS) for removing inorganic pollutants by installing lateral anodes (LA-PCPSS) using two approaches. In the first approach, the LA-PCPSS was connected to different sources of applied voltages (DSAV) from different power supplies, while in the second approach, the entire operation system was connected to the same source of applied voltage (SSAV). We used the Taguchi approach (L_9_OA) to determine the optimal levels of applied voltages for the DSAV system. The results indicated that the DSAV-(LA-PCPSS) could be optimized at an applied voltage of 1 V cm^−1^ for the surface and the first and second lateral anodes. The indigenous Sr (elements found in the tested soil without artificial pollution) in kaolinite showed the best response among other elements (Ni and other indigenous elements) when optimizing the DSAV-(LA-PCPSS) using the Taguchi approach. Installing lateral anodes (position B) supplied to low applied voltage (0.5 V cm^−1^) improved the electroosmosis (EO) rate/dewatering. Reverse migration of ions was observed during the remediation of real contaminated soil using the SSAV-(LA-PCPSS). The DSAV-(LA-PCPSS) is considered an appropriate design for the SEKR of inorganic pollutants, and increases the EO flow/dewatering. Additionally, the increased energy consumption employing the DSAV-(LA-PCPSS) was extremely minimal compared to the traditional PCPSS, which is an economic advantage for SEKR research. The DSAV-(LA-PCPSS) is still under optimization/intensification process, and subsequent processes will be examined to achieve high efficiency.

**Supplementary Information:**

The online version contains supplementary material available at 10.1038/s41598-024-75060-6.

## Introduction

Nowadays, pollution is considered a global issue that affects the soil, air, and water^1^. Pollution of the environment occurs from inorganic^2,3^, and organic pollutants^4^, as well as microplastic^5^, radioactive materials^6^, and anions^7^. Pollution can occur in a variety of ways, ranging from natural disasters to excessive chemical production, inflicting harm to plants, animals, and humans while disturbing the equilibrium of natural ecosystems^8^. Several strategies were developed to combat soil and water pollution, including chemical, physical, biological, and coupled/integrated procedures, depending on the kind of pollutant^3,8–11^.

The research on SEK began extensively in the early 1990s after the principles and basics were established by Probstein and Hicks in 1993, Acar and Alshawabkeh in 1993, Acar et al., 1993, 1995^12–15^. Since then, the SEK research has been extended to cover several areas of interest such as soil remediation, dewatering, land restoration, geophysics, sedimentation, pollution prevention, consolidation, addressing water crisis, soil nutrient availability, and seed germination^16–20^. The application of SEKR technique faced several challenges (e.g., pH variations near electrodes, formation of pH-jumping zone, occurrence of reverse electroosmosis flow, reduction of currents passing, cementation near the electrode, reduction in the electroosmosis (EO) flow rate over time, increasing temperature, occurrence of cracks in the treated soil, and formation of unsaturated zones^17,21–23^. To tackle these challenges, scientists have made significant effort and successfully enhanced the SEK method by continuously modifying the design of SEKR apparatus or adding enhancement materials^17,20,24–27^.

A review on SEK research design modification was recently published, introducing approximately 125 SEK innovative designs from worldwide scientists in various fields of interest from 1993 to 2020^17^. In this review, the SEKR design was classified based on the position of installed electrodes into horizontal, vertical, and mixed (horizontal and vertical) approaches. From 1993 to 2020, the SEKR horizontal approach involved 19 processes, the vertical approach involved 11 processes, and the mixed approach involved 7 processes, summarizing the drawbacks of SEKR during this period and how innovative research overcame these challenges^17^. The modifications made in the SEK design from 2021 to 2022 included 13 processes for the horizontal approach and 3 processes for the vertical approach, while the mixed approach (combining horizontal and vertical processes) was not presented in the literature^28^. The enhancement of SEKR using perforated electrodes, pipes, and nozzles has been recently reviewed^20^, highlighting the advantages of proper SEK implementation such as collecting drainage water, removing nitrate, injecting enhancement materials, reducing pH advection, incorporating a vacuum system, distributing water throughout treated soil, and using it as a monitoring well. Additionally, SEKR can be improved by applying reverse polarity mode^29^, pulsed electric field^30,31^, approaching/moving electrodes^32^, incorporate a vacuum^33^, avoiding crack formation^28^, continuously reorienting the electric field^34,35^, adjusting anolyte and catholyte solutions^36^, adding chemical to the target soil^27^, ultrasound/ultrasonic^37,38^, choosing the appropriate drainage position^39^, etc. The beneficial effects of electric fields are also extended to include water purification and desalination^40–42^, oxidation of organic pollutant-containing wastewater^43,44^, electrocoagulation^45^, electrodeionization^46^, and metal recovery by electrolysis/electrodeposition^47^.

Abou-Shady and Peng introduced the PCPSS in 2012^23^ as a new ex-situ electrokinetics pollutant removal process. The PCPSS’s design allows for the creation of acid water above the surface anode, which is then moved by EO to the bottom cathode, transporting the acid water through the polluted soil with inorganic pollutants. In addition, the cathode’s perforated pipe shape enables for the continuous discharge of alkaline water surrounding the bottom cathodes and gasses outside the SEKR apparatus. The PCPSS was recently investigated for reclaiming high salt-affected soils^48^. The enhancement of the PCPSS involved evaluating various positions of anodes for the removal of inorganic elements^49–51^. According to the recent classification of the SEKR^17,28^, the PCPSS belongs to the SEKR vertical design.

The main goal of the present work is to enhance the performance of the PCPSS by installing lateral anodes in the gap between the surface anode and the downward cathode pipes. Based on our published reviews during the period 1993–2023^17,20,27–29,31,32^ and recent published review and books by other scientists, no studies have investigated the effects of lateral anodes on SEKR-PCPSS^24,26,52–61^. In this study, we investigated two different LA-PCPSS approaches: (a) DSAV and (b) SSAV. With the DSAV approach, the LA-PCPSS was connected to different power supply sources to apply voltages, while with the SSAV approach, the whole system was connected to a single power supply source to apply the required voltage. Nine trial experiments were done utilizing the Taguchi technique (L_9_OA) to identify the appropriate levels of applied voltages for carrying out the DSAV’s confirmation experiment on real contaminated soil.

## Materials and methods

### SEKR apparatuses

#### The perforated cathode pipe SEKR system (PCPSS)

**Fig. 1:**
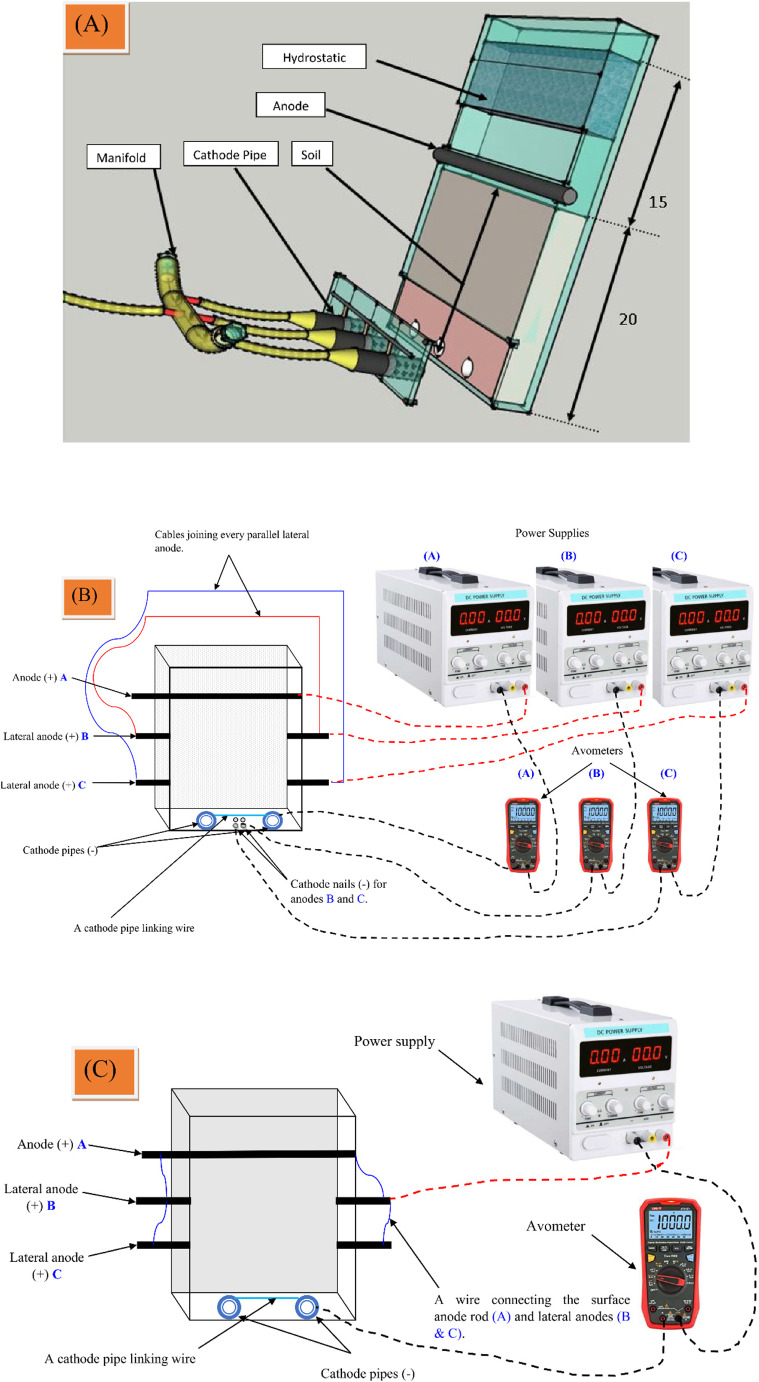
Schematic diagrams show the ideal/traditional design of the perforated cathode pipe SEKR system (PCPSS) after Abou-Shady & Peng, ; Abou-Shady^23,48^, note that this is the original design introduced in 2012, and because it represents a clear image for readers, we presented it for illustration (**A**), the lateral anodes perforated cathode pipe SEKR system (LA-PCPSS) with the different sources of applied voltages (DSAV) (**B**), and the LA-PCPSS connected with the same source of applied voltage (SSAV) (**C**).

The PCPSS design used in our previous investigations is presented in Fig. 1a^23,48^. In this study, the PCPSS was made from Acrylic in a rectangle shape with internal dimensions of 28 cm length × 24 cm width× 4 cm height, without hydrostatic. The Acrylic box was filled with polluted soil up to 20 cm from the bottom. The anode rod was made of titanium (28 cm length, 1.5 diameter) in order to prevent electrode corrosion. The cathode was composed of a stainless steel pipe owing to there is no corrosion, thus any kind of electrode material can be utilized. The cathode pipe (10 cm length, 1.5 cm diameter) provided with holes (12 holes for each pipe, spaced 3 cm apart) for the portion that was placed within the PCPSS. The holes were wrapped with two pieces of old cloth to avoid clogging. Two cathode pipes were installed with an 11 cm gap between them, and the vertical distance between the surface anode and cathode pipes was 20 cm^23^. A lateral anode (LA-PCPSS) was provided within the gap between the surface titanium anode rod and cathode pipes. To prevent water evaporation during operation, both the PCPSS and LA-PCPSS were covered with a 1 cm thick Acrylic sheet (28 cm length × 4 cm width) secured with an iron nail at the top. Furthermore, putting the acrylic cover in this manner allows for the easy supply of water, preventing dryness during the operation. The borders of the PCPSS and the LA-PCPSS were made of 2 cm thick Acrylic.

#### The different sources of applied voltages technique (DSAV-(LA-PCPSS))

The DSAV-(LA-PCPSS) is the optimal design of the PCPSS modified by including lateral anodes with external sources of applied voltages for each pair of parallel anodes, as depicted in Fig. 1b. In this approach, we investigated the installation of a couple of two pairs of lateral anodes. The first pair of anodes were fixed 6.6 ± 0.2 cm beneath the surface anode (titanium rod), while the second pair was fixed 13.4 ± 0.2 cm beneath the surface anode (6.6 ± 0.2 above the downward cathode pipes). The distance between the lateral anodes and the main anode and cathode based on the ability to install the lateral anodes in acrylic at a vertical distance of 20 cm with ease. We discovered that installing two lateral anodes allowed us to fit the most electrodes into the vertical gap between the surface anode and the bottom cathode pipe. In the primary investigation carried out using the Taguchi approach to optimize the performance of the DSAV-(LA-PCPSS), the cathodes of the lateral anodes consisted of iron nails (3 cm length, 3 mm thickness), with 2 iron nails connected as cathodes for each pair of lateral anodes. The iron nails were fixed in the middle distance between the two cathode pipes and ~ 1.5 cm of the iron nail extended inside the apparatus to be in direct contact with the polluted soil. The lateral anodes were made of graphite (~ 5 cm length × 1.5 cm in diameter) and fixed on the right and lift sides of the PCPSS (1.5 cm of the lateral anode extended inside the LA-PCPSS) to be in direct contact with the polluted soil. We chose graphite as the lateral anode material since it was easy to cut into small pieces and hence ideal for the purpose. In the confirmation experiment, the cathode iron nails were replaced with graphite electrodes (3 cm length × 1.5 in diameter) fixed inside the apparatus and connected with applied voltage by an iron nail. The connection of direct current (DC) was provided from different power supplies, as presented in Fig. 1b. Our hypothesis for the DSAV-(LA-PCPSS) approach was the installation of various lateral anodes provided with electricity from different sources of applied voltages may increase the electromigration of inorganic pollutants (removal efficiency), increase dewatering rate, and decrease the energy consumption.

#### The same source of applied voltages technique (SSAV-(LA-PCPSS))

The SSAV-(LA-PCPSS) refers to the ideal/traditional design of the PCPSS that was modified by incorporating lateral anodes and connected with the surface/top anode rod and supplied with electricity from the same source of DC power supply. In the SSAV-(LA-PCPSS), no extra cathodes were fixed in the middle distance of the cathode pipes as previously done with the DSAV-(LA-PCPSS) (Fig. 1c). Our hypothesis for the SSAV-(LA-PCPSS) approach was that the installation of different lateral anodes provided with electricity from the same source of applied voltages may increase the electromigration of inorganic pollutants, as well as it may increase the dewatering/EO rate, and reduce the energy consumption.

### The Taguchi approach

The Taguchi approach was used to determine the ideal levels of DC applied voltages needed for optimal performance of the DSAV-(LA-PCPSS). Previous studies have shown that Ni-containing contaminated soil is the most resistant to removal using PCPSS compared to other inorganic pollutants^49,62^. As a result, Ni was chosen as an inorganic pollutant representative in the primary studies conducted to determine the suitable amounts of applied voltages for improving the performance of the DSAV-(LA-PCPSS). Our hypothesis was that if we were succeeded in enhancing Ni removal using enhanced PCPSS, it would benefit the remainder of the heavy metals/inorganic pollutants due to Ni’s strong resistance to removal from contaminated soil. Nine trials were conducted using the Taguchi approach with artificially contaminated kaolinite with Ni (50 mg kg^−1^) to improve the performance of DSAV-(LA-PCPSS). Ni solution was created by dissolving NiCl_2_.6H_2_O (Analytical Reagent, 98% purity, Techno Pharmchem) in distilled water to stimulate contaminated soil. The Ni solution was thoroughly combined with kaolinite until the soil paste look was achieved. Commercial kaolinite was obtained from Green Egypt Company, 10th of Ramadan City, Egypt. The Taguchi approach was utilized as an experimental design to precisely investigate the experimental variables at different levels^63^. Table 1 lists the Taguchi technique experimental design used to improve the DSAV-(LA-PCPSS), which included the use of L_9_OA. The values signal-to-noise ratio (S/N) (Table 2) were analyzed in our experiment using the following equation based on Taguchi theory:

1 $$\text{The larger the better=-10 log}^{10}(\frac{1}{n}\sum\nolimits^{n}_{j=1}\frac{1}{SP^{2}_{j}})$$

where SP is the experimental response^40,63^.

**Table 1:** Experimental design utilizing the Taguchi approach (3 factors at 3 levels) to maximize the performance of the DSAV-(LA-PCPSS) with ni as a representation of heavy metals and other elements.

Trials nos.	The voltages applied to the DSAV-(LA-PCPSS), a connection between the surface anode rod and bottom cathode pipes, (position A).	The voltages applied to the DSAV-(LA-PCPSS), a connection between the first lateral anodes (located 6.6 ± 0.2 cm beneath the surface anode and 13.4 ± 0.2 cm above the bottom cathode nails) and bottom cathode nails (position B).	The voltages applied to the DSAV-(LA-PCPSS), a connection between the second lateral anodes (located 13.4 ± 0.2 cm beneath the surface anode and 6.6 ± 0.2 cm above the bottom cathode nails) and bottom cathode nails (position C).
1	0.5 V cm^−1^ (10 V)	0 V cm^−1^ (0 V) “no lateral anodes”	0 V cm^−1^ (0 V) “no lateral anodes”
2	0.5 V cm^−1^ (10 V)	0.5 V cm^−1^ (6.6 V)	0.5 V cm^−1^ (3.3 V)
3	0.5 V cm^−1^ (10 V)	1.0 V cm^−1^ (13.3 V)	1.0 V cm^−1^ (6.6 V)
4	1.0 V cm^−1^ (20 V)	0 V cm^−1^ (0 V)	0.5 V cm^−1^ (3.3 V)
5	1.0 V cm^−1^ (20 V)	0.5 V cm^−1^ (6.6 V)	1.0 V cm^−1^ (6.6 V)
6	1.0 V cm^−1^ (20 V)	1.0 V cm^−1^ (13.3 V)	0 V cm^−1^ (0 V)
7	1.5 V cm^−1^ (30 V)	0 V cm^−1^ (0 V)	1.0 V cm^−1^ (6.6 V)
8	1.5 V cm^−1^ (30 V)	0.5 V cm^−1^ (6.6 V)	0 V cm^−1^ (0 V)
9	1.5 V cm^−1^ (30 V)	1.0 V cm^−1^ (13.3 V)	0.5 V/cm (3.3 V)

**Table 2:** The removal of Ni (%) from the first, second, third, fourth, and top four layers together and its corresponding signal-to-noise (S/N) ratio.

Trials nos.	1^st^ layer		2^nd^ Layer		3^rd^ layer		4^th^ layer		Top four layers	
	Removed Ni (%)	S/*N* ratio	Removed Ni (%)	S/*N* ratio	Removed Ni (%)	S/*N* ratio	Removed Ni (%)	S/*N* ratio	Removed Ni (%)	S/*N* ratio
1	90.38	39.12	-12.78	22.13	-42.33	32.53	-4.03	12.10	7.81	17.86
2	91.81	39.26	-8.23	18.31	-48.82	33.77	1.53	3.68	9.07	19.15
3	59.83	35.54	3.75	11.48	13.54	22.63	2.33	7.33	19.86	25.96
4	92.40	39.31	-15.17	23.62	-27.64	28.83	-33.78	30.57	3.95	11.93
5	60.41	35.62	-22.20	26.93	8.24	18.31	0.75	-2.48	11.80	21.44
6	42.90	32.65	22.37	26.99	-6.32	16.01	3.26	10.25	15.55	23.84
7	47.50	33.53	-6.40	16.12	2.81	8.97	8.93	19.02	13.21	22.42
8	56.15	34.99	-67.00	36.52	2.06	6.27	1.06	0.48	-1.93	5.73
9	63.18	36.01	4.86	13.73	-19.19	25.66	-3.77	11.53	11.27	21.04

Table 1 shows the researched factors (three applied voltages) at three levels. Different applied voltages levels were recommended, representing low electric potential (0.5 V cm^−1^), high electric potential (1.5 V cm^−1^), and optimal electric potential (1 V cm^−1^) for SEKR operation. Based on our previous published reviews^17,28^, we selected voltage gradient levels that were above and below the ideal value of 1 V cm^−1^. These values varied depending on the vertical distance between the surface and lateral anodes and the bottom cathodes. The applied voltages to surface anode and cathode pipes (position A) were 0.5 V cm^−1^ (10 V), 1 V cm^−1^ (20 V), and 1.5 V cm^−1^ (30 V). The voltages applied to the first set of lateral anodes and cathode nails (position B) were zero (zero V), 0.5 V cm^−1^ (6.6 V), and 1 V cm^−1^ (13.3 V), with zero voltage indicating that no lateral anodes were installed. The applied voltages for the second pair of lateral anodes (position C) were zero (zero V, no lateral anodes), 0.5 V cm^−1^ (3.3 V), and 1 V cm^−1^ (6.6 V), as listed in Table 1.

The kaolinite was utilized as a representative of porous media (electrical conductivity = 3.5 dS m^−1^; 1:5 extract), with a pH of 7.73 (soil paste extraction). Other indigenous elements found in the kaolinite include Sr (13.4 mg kg^−1^), Zn (**36.5** mg kg^−1^), Pb (**9.4** mg kg^−1^), Cr (14.4 mg kg^−1^), Cu (11.1 mg kg^−1^), and Mn (17.4 mg kg^−1^). The power supplies (RXN-305D, 0–30 V, made in China) and (RXN-3010 30–60 V, made in China) were utilized to deliver stable DC. An electrical converter (TDGC_2_-0.05 and TDGC_2_-1, made in China) was also utilized to provide a power supply, with a special bridge connected to convert the alternating current (AC) to DC^17^. An avometer (UT61E, made in China) was connected to the electric circle to precisely measure the current passing through the PCPSS. The nine trials were conducted using the Taguchi approach via dividing it into two groups: the first group involved the simultaneous operation of trials 1–4, while the second group featured the simultaneous operation of trials 5–9. The nine trials lasted for 30 days. In the first group, kaolinite was observed leaking from the cathode pipes in trial 4, whereas this was the predominant feature of the second group’s trials. In second group of trials (5–9), it was detected a reduction of kaolinite volume inside the DSAV-(LA-PCPSS), the establishment of a tiny gap between the surface anode and kaolinite during the first 2 days of operating was observed. This could be attributed to the escaping kaolinite from the cathode pipes. To address this issue, pure kaolinite was poured from the top of the DSAV-(LA-PCPSS) to fill the gap and assure an electric connection. Throughout the 30-day experiment period, 100–200 ml of tap water (pH = 7.2, Ec = 358 µS cm^−1^) was manually injected above the surface anode rod every 2–3 days to maintain the kaolinite moist.

### Confirmation experiments

After the investigation the potential of lateral anode installation within the ideal/traditional design of the PCPSS using the DSAV-(LA-PCPSS), we conducted confirmation experiments using real contaminated soils collected from the surface and subsurface layers of the El-Gabal El-Asfar area, Egypt. The properties of real contaminated soil were as follows: EC (1.2 dS m^−1^), pH (7.2), texture (Sandy Clay Loam), organic matter (2.07%), and CaCO_3_ (0.70%). The hydraulic conductivity for the real contaminated soil was 0.0035 cm h^−1^ which is considered very low according to USDA classification^64^. The mean concentrations of the total content of heavy metals and other elements in the real contaminated soils were as follows: Ni (33.5 mg kg^−1^), Zn (522.2 mg kg^−1^), Pb (205.3 mg kg^−1^), Cd (0.8 mg kg^−1^), Cr (255.1 mg kg^−1^), Cu (160.45 mg kg^−1^), Sr (118.1 mg kg^−1^), and Mn (236.8 mg kg^−1^).

The total concentrations of heavy metals in the soils were detected using the ICP (iCAP PRO X Duo – Thermo Fisher) after digestion using a digestion mixture^65^. The concentration ratio (CR) was used to evaluate the behavior of inorganic elements under the applications of different designs of the SEKR-PCPSS. The CR could be calculated as follows:

2 $$\:\text{C}\text{R}=\:\frac{The\:concentration\:of\:ion\:after\:treatment}{\:\:The\:concentration\:of\:ion\:before\:treatment}$$

## Results and discussion

### Optimizing the removal efficiency of the DSAV-(LA-PCPSS) using the Taguchi approach

The performance of the DSAV-(LA-PCPSS) was optimized using the Taguchi approach (L_9_OA), which comprises three factors at three levels (Tables 1 and 2) to choose the appropriate values of applied voltages for the different positions of electric connections (position A, position B, and position C), as shown in Fig. 1b. Ni was chosen as a representative for heavy metals and other elements because our previous studies revealed its high resistance to the SEKR^49,62^. If we can enhance the remediation of Ni using the SEKR approach, removing the rest of the heavy metals and other elements will be improved simultaneously. To ensure the ideal circumstances for SEKR, commercial kaolinite was used to represent the ideal porous media and low-permeability soil. The commercial kaolinite contains indigenous concentrations of Sr, Zn, Pb, Cr, Cu, and Mn that were also evaluated after terminating the DSAV-(LA-PCPSS) trials.

#### Effect of different voltage sources on the removal of Ni from different layers utilizing the DSAV-(LA-PCPSS)

**Fig. 2:**
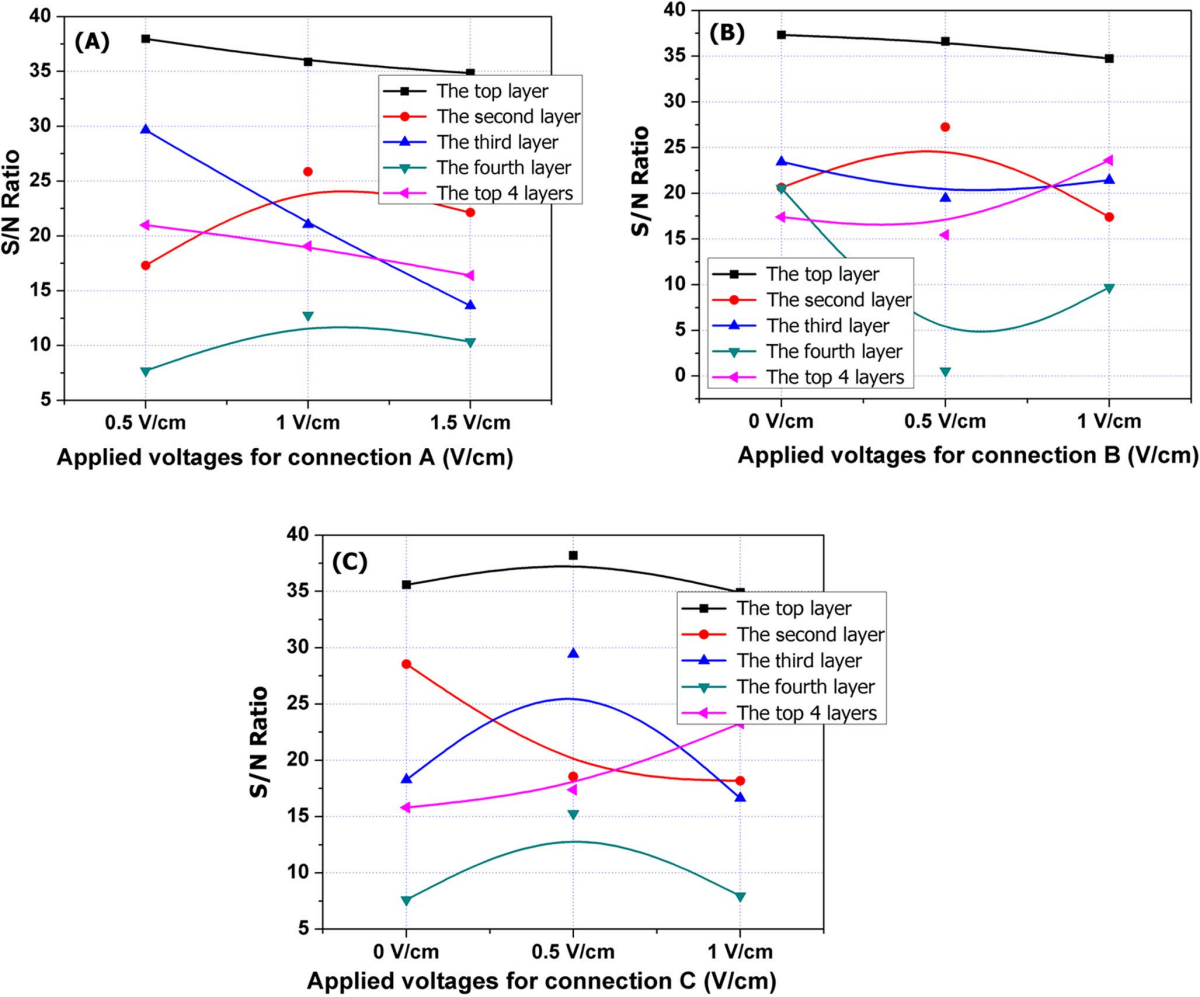
Effect of applied voltages on Ni removal efficiencies from the top four layers utilizing the DSAV-(LA-PCPSS).

In this section, we looked at how different applied voltages using the three-position connection (A, B, and C) proposed for the DSAV-(LA-PCPSS) affect the removal of Ni. We used the Taguchi approach (L_9_OA) to investigate this. The polluted soil containing the SEKR apparatus was divided into five main layers, each 4 cm thick. We examined the removal of Ni for the top four layers, excluding the fifth layer due to continuous element accumulation and relatively high pH near the cathode pipes or nails. Figure 2a shows the effect of different applied voltages connected to position A (the connection between the surface anode rod and the bottom cathode pipes) on Ni removal percentages. We found that the highest removal of Ni in the first layer was achieved with the lowest value of applied voltage of 0.5 V cm^−1^, and increasing the applied voltages from 0.5 to 1 V cm^−1^ resulted in a slight reduction in Ni removal percentages. Elevating the applied voltage from 1 V cm^−1^ to 1.5 V cm^−1^ also caused a slight reduction in Ni removal percentages. This finding may be convenient with our hypothesis that installing the lateral anodes may improve the removal rates of inorganic pollutants from different layers; thus, elevating the applied voltages that are considered the driving force of electromigration to enhance the removal efficiency is not necessary. This will eventually minimize the energy usage for the electric connection of position A. In the second layer, increasing the applied voltage from 0.5 V cm^−1^ to 1 V cm^−1^ resulted in the optimal removal of Ni, but further increases from 1 to 1.5 V cm^−1^ caused a reduction (Fig. 2a). In the third layer, the Ni removal rate was reduced by increasing the applied voltage from 0.5 V cm^−1^ to 1 V cm^−1^ and then to 1.5 V cm^−1^. The removed Ni from the fourth layer followed the same pattern as Ni removal in the second layer, where the highest removal rate was seen with an applied voltage of 1 V cm^−1^. The removal percentages of the sum of the top four layers followed the same tendency of the first layer. Based on data derived from Fig. 2a, it was concluded that an applied voltage of 1 V cm^−1^ may achieve the best removal rate for either the second or fourth layers, while a minor reduction was accomplished in the first layer and the sum of the top four layers. Previous reviews on enhanced SEKR by design modifications and chemical additives recommended an applied potential of 1 V cm^−1^ for the SEKR^17,20,27,28^. As a result, we chose an applied voltage of 1 V cm^−1^ for position A to proceed with the confirmation experiment using real contaminated soils with the DSAV-(LA-PCPSS).

Figure 2b illustrates the impact of applied voltages of lateral anodes located in position B on the removal percentages of Ni from the top four layers (the applied voltage of zero refers to no lateral anodes installed). In the first layer, there was a very minimal reduction (neglectable) in the removed Ni when an applied voltage of 0.5 V cm^−1^ was connected to the lateral anodes (position B). Increasing the applied voltage to from 0.5 to 1 V cm^−1^ resulted in a slight reduction in removed Ni. In the second layer, connecting the electricity at 0.5 V cm^−1^ increased the removal percentages compared to zero voltage mode, while an increase to 1 V cm^−1^ decreased the removal percentages. In the third layer, connecting the applied voltage of 0.5 V cm^−1^ slightly decreased the removal percentage of Ni compared to the zero voltage, but further increases from 0.5 to 1 V cm^−1^ improved it.

The effect of applied voltages of a lateral anode of position B on the removed Ni from the fourth layer followed a similar trend observed with the third layer, with a sharp reduction seen with an applied voltage of 5 V cm^−1^. The effect of applied voltages connected to the lateral anodes of position B on the total removal of Ni from the top four layers was almost the same with applied voltages of 0 and 0.5 V cm^−1^, but the removal percentages noticeably improved when the applied voltage 1 V cm^−1^ was connected. Therefore, an applied voltage of 1 V cm^−1^ for position B was chosen to proceed with the confirmation experiment for removing heavy metals and other elements from real contaminated soils using the DSAV-(LA-PCPSS).

Data depicted in Fig. 2c reveal that the connecting of electricity to the lateral anodes located in position C with 0.5 V cm^−1^ improved the removal percentages of Ni in the first layer compared with the applied voltage of 1 V cm^−1^. The same tendency was also observed for the removed Ni from the third and fourth layers. In the second layer, there was a sudden reduction in the removed Ni when the applied voltage was 0.5 V cm^−1^ and there was no change in the removal rate when the applied voltage was increased from 0.5 V cm^−1^ to 1 V cm^−1^. In general, the total removal of Ni from the top four layers was enhanced when the applied voltage was increased from 0.5 V cm^−1^ to 1 V cm^−1^ (Fig. 2c). Therefore, the applied voltage of 1 V cm^−1^ for position C was chosen to proceed with the confirmation experiment of the DSAV-(LA-PCPSS). To summarize past findings, the applied voltage of 1 V cm^−1^ was chosen to proceed with the confirmation experiments of the DSAV-(LA-PCPSS) for different anode positions: position A (ideal/traditional design of PCPSS), position B (the first lateral anodes), and position C (the second lateral anodes) using real contaminated soils with inorganic pollutants.

####  Distribution of Ni and indigenous elements inside the DSAV-(LA-PCPSS) apparatus following Taguchi trials

**Fig. 3:**
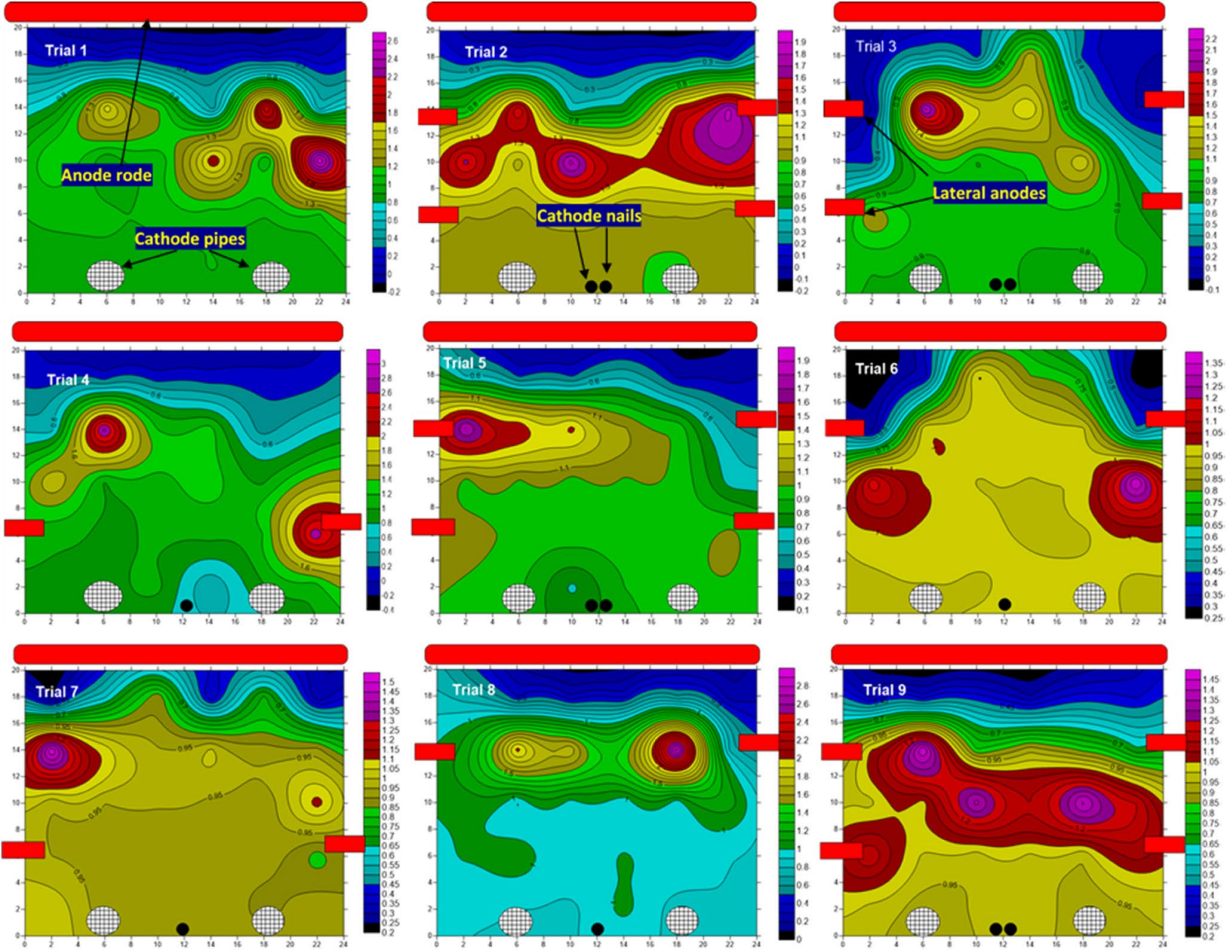
Distribution of Ni within the DSAV-(LA-PCPSS) over the nine trials proposed by the Taguchi approach.

In this section, we evaluated the distribution of Ni and other indigenous elements (Sr, Zn, Pb, Cr, Cu, and Mn) in the DSAV-(LA-PCPSS) apparatus after ending the Taguchi trials. Figure 3 shows the Ni distribution in the 9 trials conducted based on the experimental design proposed by the Taguchi approach. We observed that, when the DSAV-(LA-PCPSS) was performed without installing the lateral anodes (ideal/traditional design of the PCPSS, trial 1) using applied voltages of 0.5 V cm^−1^ (~ half of the ideal applied voltage), Ni was removed from the first and second layers and tended to accumulate in the subsequent layers. Similar tendencies were observed in trials 2, 4, 5, 8, and 9. However, in trials 2 and 9, the intensity of accumulation of Ni in the third layer was more pronounced. In trials 3, 6, and 7, Ni tended to migrate towards the anode in the DSAV-(LA-PCPSS). Overall, the removed Ni in the 9 trials was unclear, possibly due to its high resistance to remediation compared with other heavy metals and other elements using the SEKR-PCPSS^49,62^.

The removal of indigenous Sr, as shown in Fig. S1 indicated the best response for installing the LA-PCPSS using the DSAV approach. In the first trial (ideal/traditional design of the PCPSS), the migration of Sr above the cathode pipes was lower than that existing above the cathode gap, which is consistent with our previous findings^23^. In the PCPSS, ions directly above the cathode pipes are under one pulling force, whereas they will be under two pulling forces in the area above the cathode gap. This behavior also observed in trials 1, 2, 4, 5, 6, 7, 8, and 9. The best removal rate of Sr was observed in the third trial, whereas, accumulation of Sr was observed in the bottom layer in the gap between cathode pipes, possibly due to the installation of cathode nails that did not allow the discharge of the EO flow outside the DSAV-(LA-PCPSS) simultaneously with the prevailing of high pH in the bottom layer.

Figure S2 depicts the distribution of indigenous Zn within the DSAV-(LA-PCPSS) for several trials proposed by the Taguchi approach. In trials 6 and 7, Zn removal tended to collect on the anode rods. In trials 3, 5, 8, and 9, indigenous Zn tended to collect around the cathode iron nails rather than the cathode pipes. The third trial had the highest Zn removal rate of any of the nine trials.

The indigenous Pb removal rate improved in trials 3, 4, 6, 7, and 8 compared to the other trials (Fig. S3). Trials 2, 5, and 9 showed irregular removal of Pb. The increase of indigenous Pb near the lateral anodes was seen in the first lateral anodes of trials 2, 3, 5, 6, 8, and 9, confirming the presence of reverse migration. The data in Fig. S4 indicate the reaction of indigenous Cr inside the DSAV-(LA-PCPSS) for the nine trials proposed by the Taguchi approach. The highest Cr removal rates were recorded in trials 2, 5, and 8. Cr accumulation was osculated close to the cathode iron nails and cathode pipes. Fig. S5 shows the removal of indigenous Cu. Cu was shown to be regularly removed from the first layer in trials 1, 2, 4, and 9. Cu accumulation at the zone close to the surface anode rod was observed in trials 3, 5, 6, 7, and 8, showing Cu reverse migration. Trials 2, 4, 5, 6, 7, 8, and 9 showed increasing Cu accumulation close to the lateral anodes. This could be explained by the fact that the current passing through the lateral anode circuit was inverted in some experiments, causing inconsistency. Fig. S6 depicts the response of indigenous Mn within the DSAV-(LA-PCPSS) for the nine trials proposed by the Taguchi approach. The best Mn removal results were reported in trials 4, 8, and 9. Mn accumulation around the cathode iron nails was seen in trials 4, 5, 6, 8, and 9. Mn accumulation close to the surface anode rod was detected in trials 1, 2, 3, 4, 5, and 6.

#### Analysis of variance and contribution percentages

**Fig. 4:**
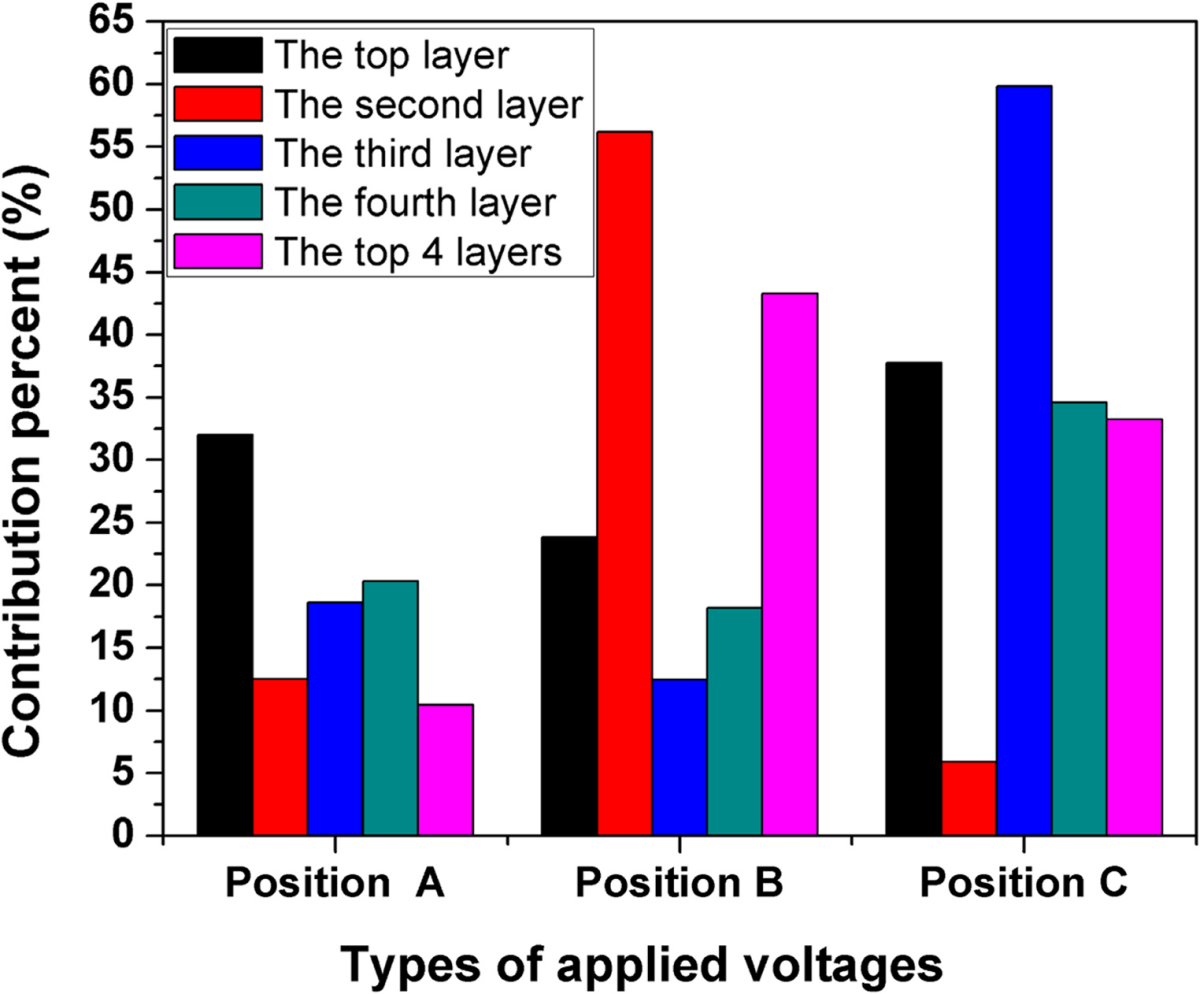
The contribution percentages of different voltages connections for Ni removal utilizing the DSAV-(LA-PCPSS) over the nine trials proposed by the Taguchi approach.

Tables 3, 4, 5, 6 and 7 show the analysis of variance for the studied applied voltages at different positions (A, B, and C) for Ni removal utilizing the DSAV-(LA-PCPSS) for the nine trials provided by the Taguchi approach. The critical values of F distribution at α (risk) levels of 0.05 and 0.01 were 3.4 and 5.6, respectively. The effects of applied voltages of positions A, B, and C on the removed Ni from the top layer were significant at α (risk) levels of 0.05 and 0.01. The effects of applied voltages of positions A, B, and C on the removed Ni from the second, third, fourth, and top four layers were insignificant, except for the effect of applied voltage of positions C on the removed Ni from the third layer at α (risk) levels of 0.05 and 0.01. The significance of any factor is determined by comparing the F values obtained from the Analysis of Variance (ANOVA) to the calculated values in the Table of Critical Values for the F Distribution. If your obtained F value is equal to or greater than the critical F-value, your result is statistically significant at that level of probability. Figure 4 shows the contribution percentages of the three anode positions (surface and lateral anodes) for the removed Ni from different layers. The first connection (ideal connection, position A) was shown to be more effective for removing Ni from the top layer, followed by the 4^th^ layer > 3^rd^ layer > 2^nd^ layer > top 4 layers. However, the contribution percentages of the applied voltages in case of connection in position B (the first lateral anodes) were found to take the following sequence: 2^nd^ layer > top 4 layers > 1^st^ layer > 4^th^ layer > 3^rd^ layer. The contribution percentages of the applied voltages for the connection in position C were in following order: 3^rd^ layer > 1^st^ layer > 4^th^ layers > top 4 layers > 2^nd^ layer.

**Table 3:** Analysis of variance performed for the removed ni from the 1^st^ layer (top layer) using the DSAV-(LA-PCPSS) over nine trials suggested by the Taguchi approach.

**Factors**	**DOF**	**SS**	**V**	**F**	**P**
Applied voltage A	2	959.0	479.5	5.0	32.0
Applied voltage B	2	713.8	356.9	3.7	23.8
Applied voltage C	2	1130.4	565.2	5.8	37.7
Error	2	191.6	95.8		
Total	8	2994.96

**Table 4:** Analysis of variance performed for the removed ni from the 2^nd^ layer using the DSAV-(LA-PCPSS) over nine trials suggested by the Taguchi approach.

Factors	DOF	SS	V	F	*P*
Applied voltage A	2	611.3	305.6	0.49	12.4
Applied voltage B	2	2748.4	1374.2	2.21	56.1
Applied voltage C	2	289.9	144.9	0.23	5.9
Error	2	1243.4	621.7		
Total	8	4893.208

**Table 5:** Analysis of variance performed for the removed ni from the 3^rd^ layer using the DSAV-(LA-PCPSS) over nine trials suggested by the Taguchi approach.

Factors	DOF	SS	V	F	*P*
Applied voltage A	2	758.4	379.2	2.05	18.6
Applied voltage B	2	507.8	253.9	1.37	12.4
Applied voltage C	2	2436.7	1218.3	6.59	59.8
Error	2	369.4	184.7		
Total	8	4072.5

**Table 6:** Analysis of variance performed for the removed ni from the 4^th^ layer using the DSAV-(LA-PCPSS) over nine trials suggested by the Taguchi approach.

Factors	DOF	SS	V	F	*P*
Applied voltage A	2	245.8	122.9	0.75	20.3
Applied voltage B	2	220.2	110.1	0.67	18.2
Applied voltage C	2	418.1	209.0	1.28	34.5
Error	2	324.7	162.3		
Total	8	1209.06

**Table 7:** Analysis of variance performed for the EO flow using the DSAV-(LA-PCPSS) over nine trials suggested by the Taguchi approach.

**Factors**	**DOF**	**SS**	**V**	**F**	**P**
Applied voltage A	2	34.2	17.1	0.8	10.4
Applied voltage B	2	141.9	70.9	3.3	43.3
Applied voltage C	2	108.9	54.4	2.5	33.2
Error	2	42.6	21.3		
Total	8	327.8			

#### A cumulative electroosmotic flow and current passing during DSAV-(LA-PCPSS) operation utilizing Taguchi’s nine-trial technique

**Fig. 5:**
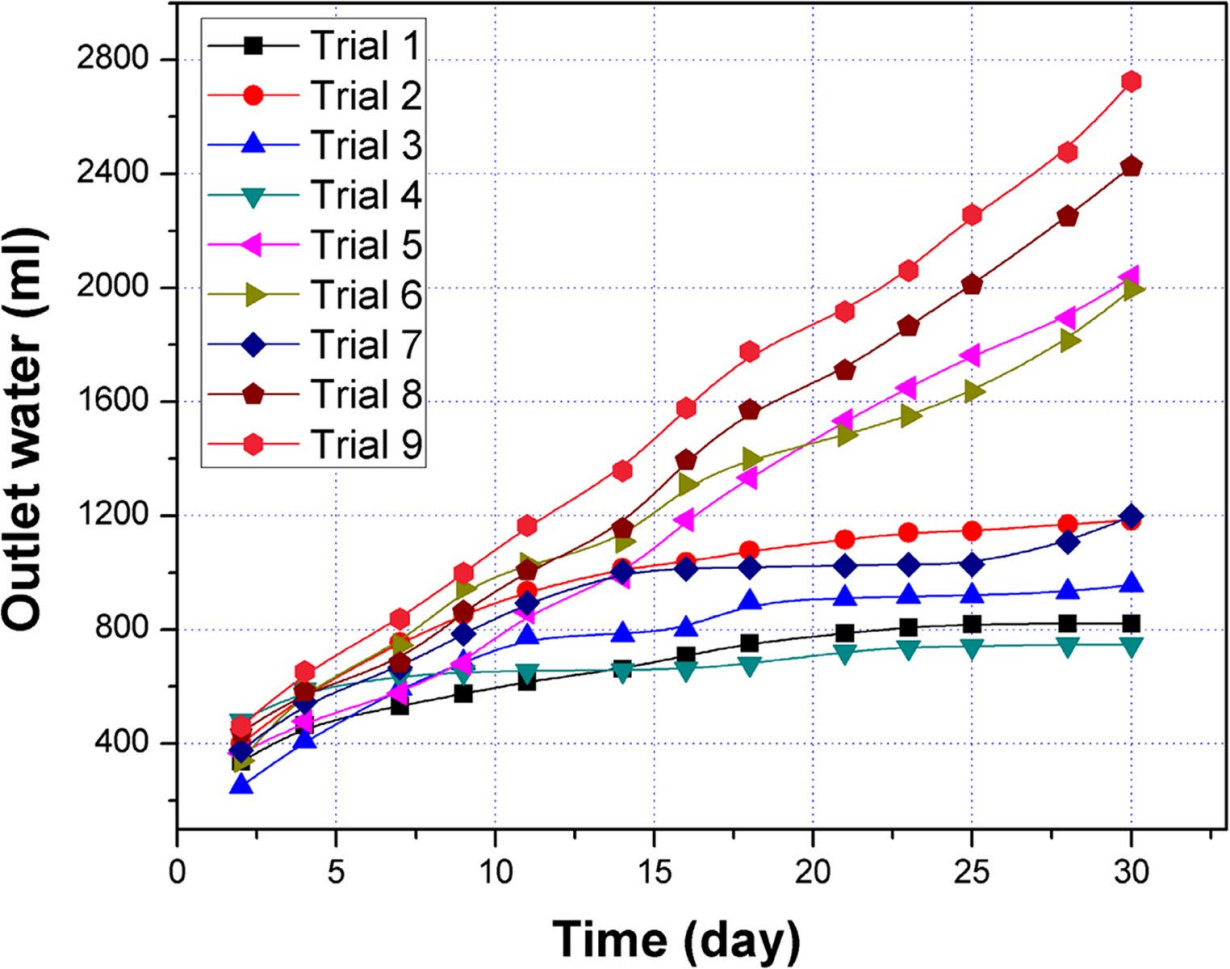
The accumulative electroosmotic flow of the DSAV-(LA-PCPSS) over the nine trials proposed by the Taguchi approach.

The EO flow of the DSAV-(LA-PCPSS) during the nine trials suggested by the Taguchi approach is shown in Fig. 5. The descending order of the EO rate was as follows: trial 9 > trial 8 > trial 5 > trial 6 > trial 2 = trial 7 > trial 3 > trial 1 > trial 4.

**Fig. 6:**
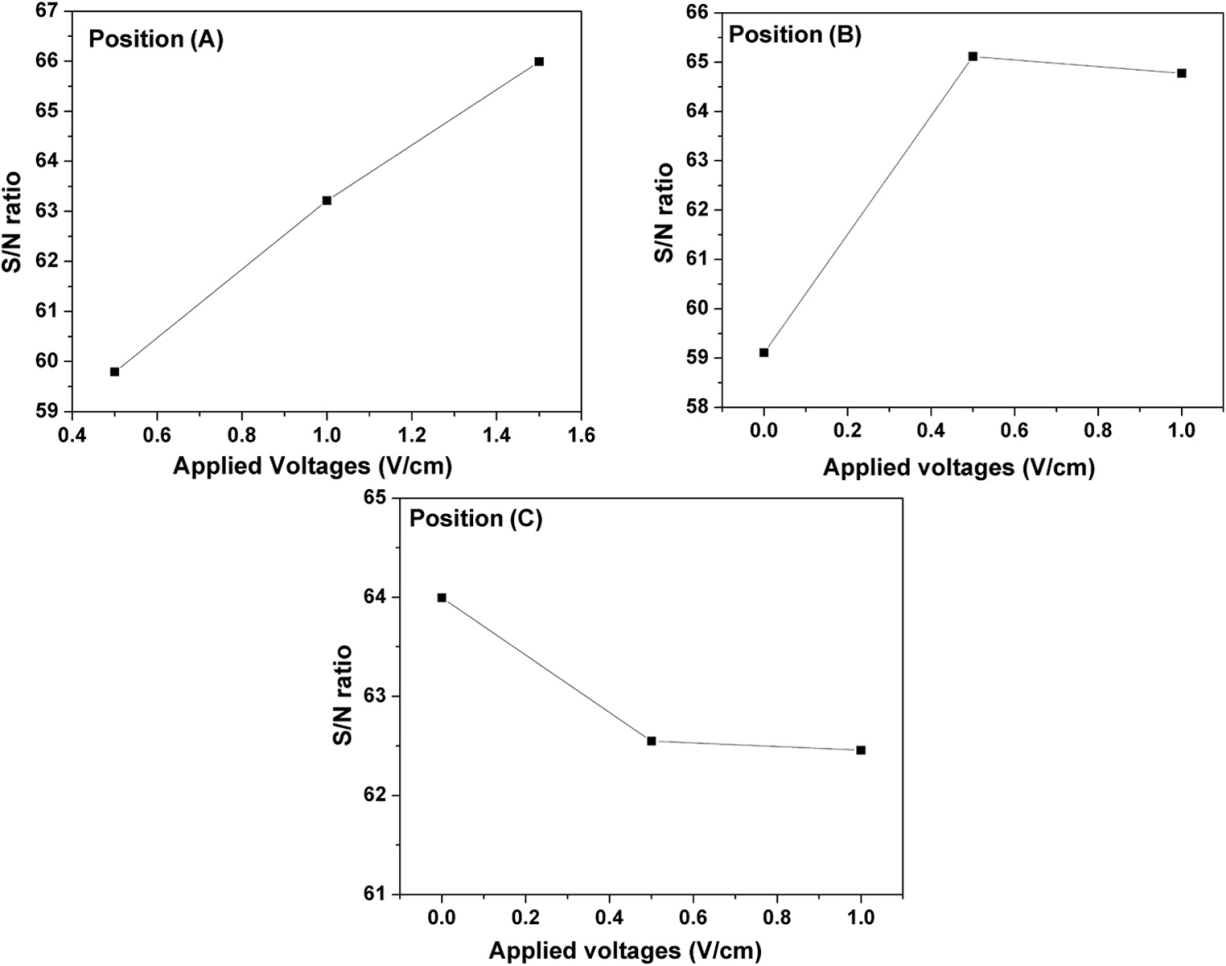
The effects of applied voltages on the EO rate for various connection types (position A, position B, and position C).

The S/N ratio indicated that increasing the applied voltages raised the EO rate for the ideal connection of the PCPSS (position A) in the sequence 1.5 V cm^-1^ > 1 V cm^-1^> 0.5 V cm^-1^ (Fig. 6). Connecting the lateral anodes with 0.5 V cm^-1^ for position B (the first lateral anodes) noticeably increased the EO rate, while increasing the voltage to 1 V cm^-1^ did not result in further improvement. While the applied voltages (0.5 and 1 V cm^-1^) were connected to position C (the second lateral anodes), the opposite behavior was observed for the ideal connection (position A). Further increase in applied voltages (from 0.5 to 1 V cm^-1^) did not lead a noticeable change in the EO flow. This observation is considered a beneficial for the electrokinetics dewatering studies, where lateral anodes may be installed in position B and supplied with a low applied voltage that ultimately may increase the amounts of EO rate in the outlet water. 

**Fig. 7:**
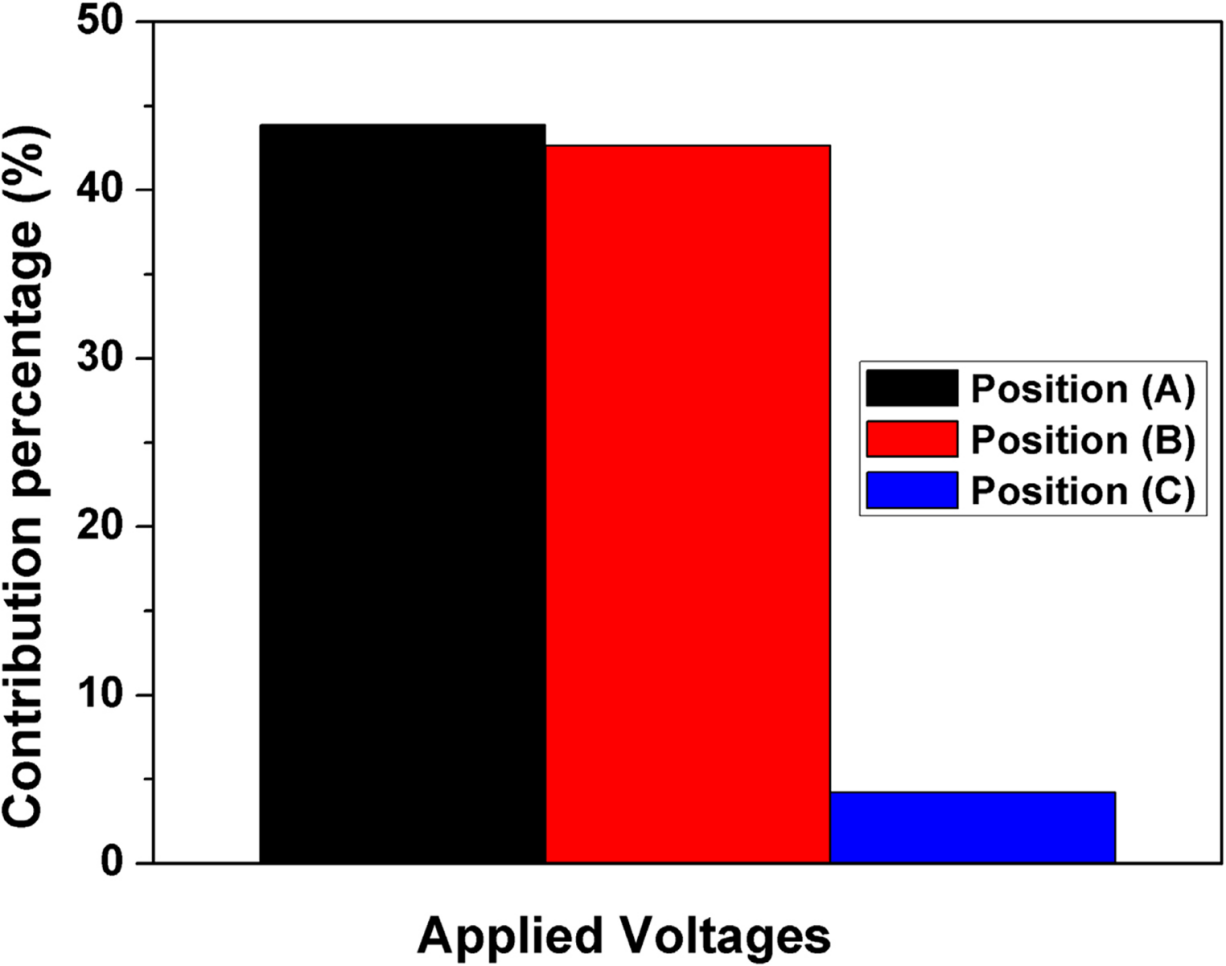
The contribution percentages of applied voltages for different connections (position A, position B, and position C) for improving the EO rate/dewatering.

**Fig. 8:**
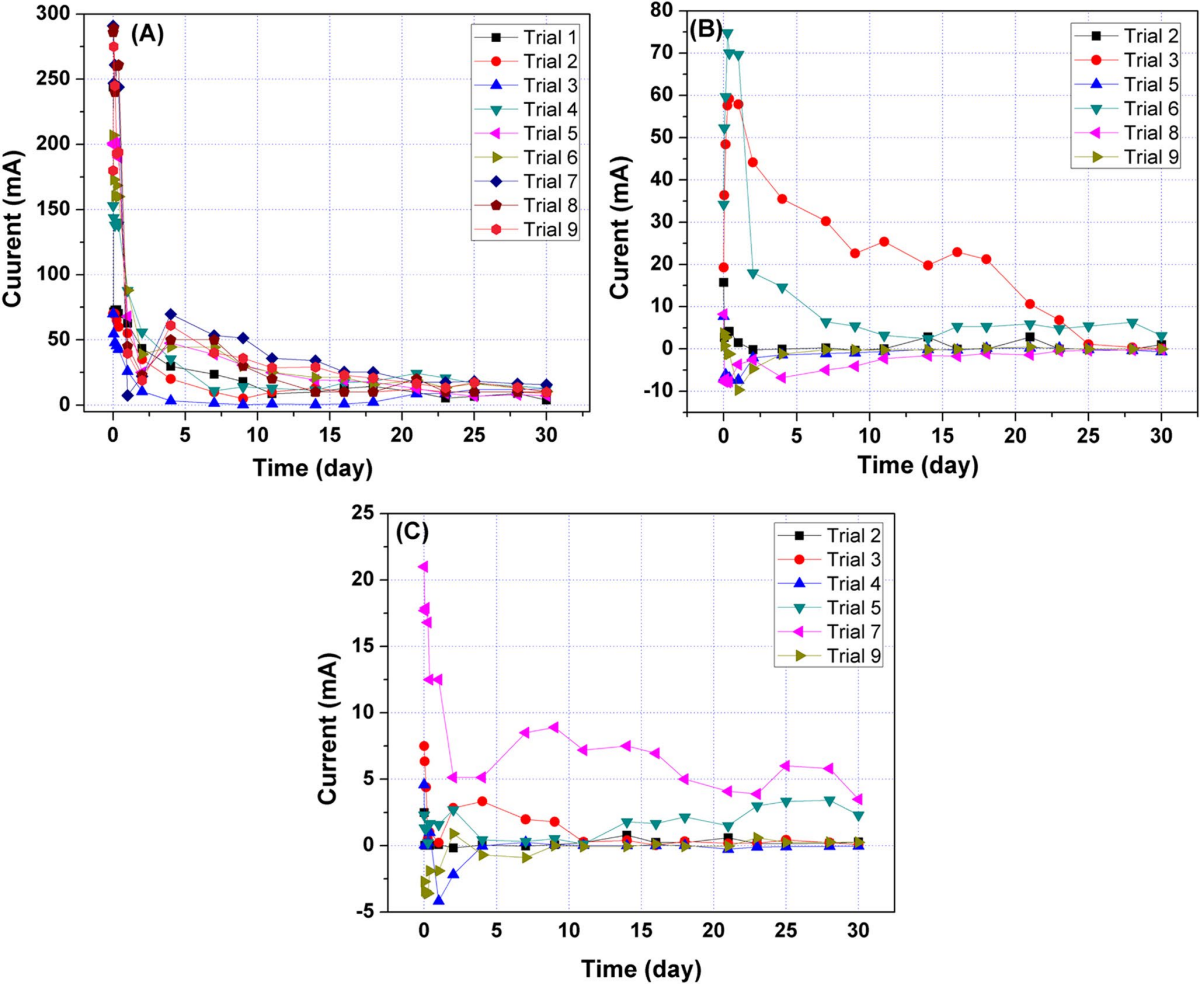
The current passing though the DSAV-(LA-PCPSS) in the nine trials proposed by the Taguchi approach; (**a**) represents the current passing through the surface anode rod and downward cathode pipes, (**b**) represents the current passing through the 1^st^ lateral anodes and downward cathode nails, and (**c**) represents the current passing through the 2^nd^ lateral anodes and downward cathode nails.

The contribution percentages of applied voltages of different connections (position A, position B, and position C) for improving the EO rate are depicted in Fig. 7. The descending order of the effect of applied voltages on improving the EO flow rate was as follows: applied voltage A > applied voltage B > applied voltage C. Table 8 displays the analysis of variance for the EO flow using the DSAV-(LA-PCPSS) based on the nine trials suggested by the Taguchi approach. At α risk of 0.05, the effects of applied voltages at position A and B were significant, while the effect of applied voltage at position C was not significant. At α risk level of 0.01, the effects of applied voltages at all three positions were not significant. In the ideal case of the PCPSS at position A, the current passing variations showed the highest values at the beginning of operation. After 22 days, the current passing variations for the nine trials were close to each other (Fig. 8). The highest current values were observed in trials 7 and 9, while the lowest values were observed in trial 3. The current passing values for the first and second lateral anodes were relatively low compared to the ideal connection at position A, likely due to the small portions of lateral anodes installed inside the LA-PCPSS (~ 1.5 cm). Reverse current in the first lateral anodes (position B) was observed in trials 5, 8, and 9, and a similar trend was observed with the second lateral anodes (position C) in trials 4 and 9. The highest current values in the first lateral anodes (position B) were observed in trials 3 and 6, while the highest values of current in the second lateral anodes (position C) were observed in trial 3, 5, and 7.

**Table 8:** Analysis of variance performed for the EO flow using the DSAV-(LA-PCPSS) over nine trials suggested by the Taguchi approach.

**Factors**	**DOF**	**SS**	**V**	F	P
Applied voltage A	2	1915874	957937.2	4.7	43.8
Applied voltage B	2	1863877	931938.5	4.6	42.6
Applied voltage C	2	184032.1	92016.03	0.4	4.21
Error	2	404654.1	202327		
Total	8	4368438			

### Confirmation experiments (comparison of the PCPSS, the SSAV-(LA-PCPSS), and the DSAV-(LA-PCPSS)

#### Remove inorganic contaminants (Ni, Zn, Pb, Cd, Cr, Cu, Sr and Mn) from real contaminated soil 

Confirmation experiments were conducted to assess the performance of different designs proposed for the LA-PCPSS using real soil contaminated with heavy metals. Five design perspectives were evaluated including: (a) the ideal/traditional design of the PCPSS (running with 1 V cm^−1^), (b) Design 1 refers to the SSAV-(LA-PCPSS) incorporated with 1 pair of lateral anodes located 6.6 ± 0.2 cm beneath the surface anode rod and connected to the same source of the applied voltage, Design 2 refers to the SSAV-(LA-PCPSS) incorporated with 2 pairs of lateral anodes located 6.6 ± 0.2 and 13.4 ± 0.2 cm beneath the surface anode rod and connected to the same source of applied voltage, Design 3 refers to the DSAV-(LA-PCPSS) incorporated with 1 pair of lateral anodes located 6.6 ± 0.2 cm beneath the surface anode rod and connected to different sources of applied voltage, and Design 4 refers to the DSAV-(LA-PCPSS) incorporated with 2 pairs of lateral anodes located 6.6 ± 0.2 and 13.4 ± 0.2 cm beneath the surface anode rod and connected to different sources of applied voltage. Eight elements of inorganic pollutants (Ni, Zn, Pb, Cd, Cr, Cu, Sr, and Mn) were detected after terminating the SEKR experiments to evaluate the above-mentioned designs of the SEKR.

**Fig. 9:**
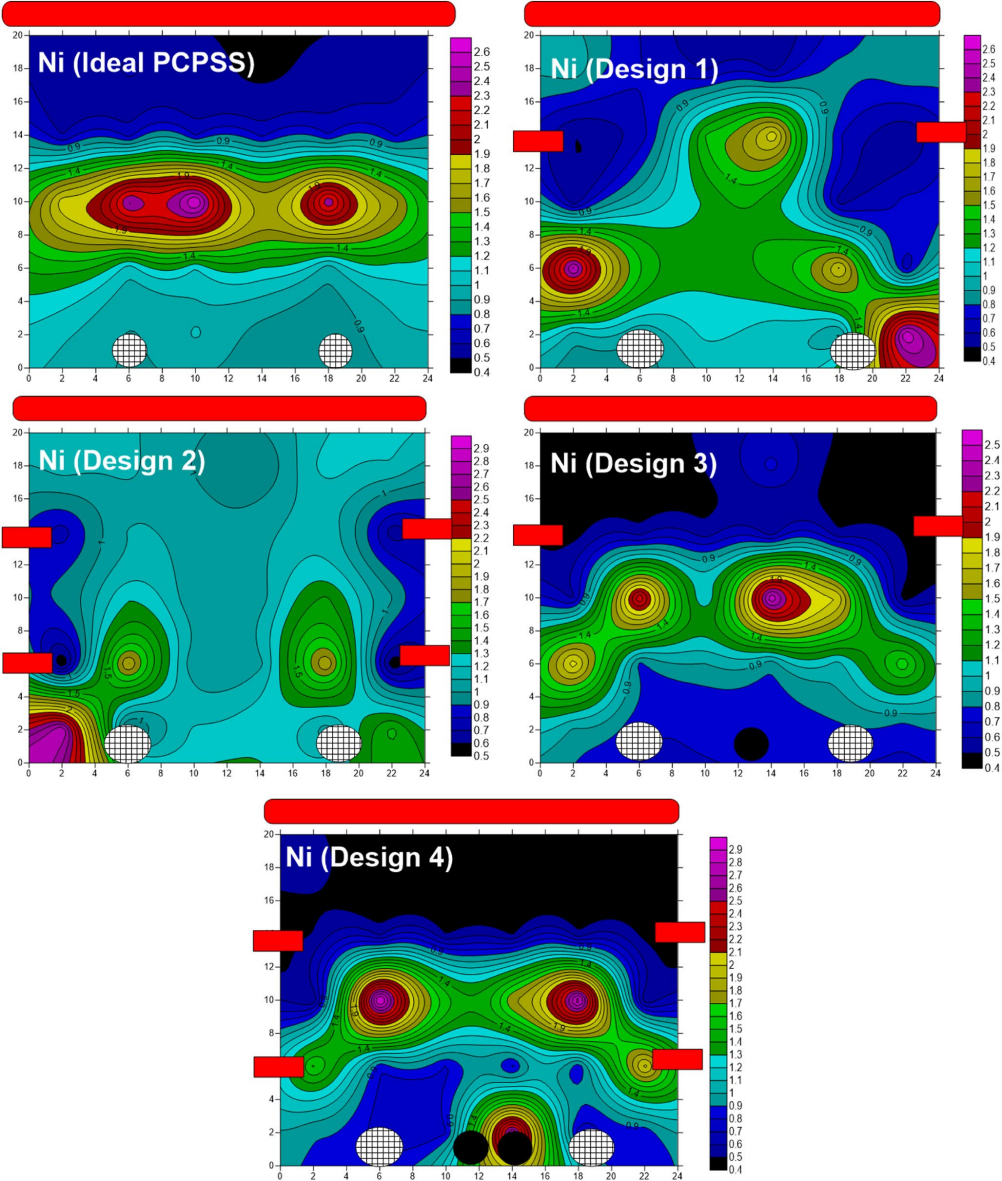
The removal of Ni from real contaminated soil using the ideal/traditional and improved designs of the PCPSS. Design 1 of the SSAV-(LA-PCPSS) has a pair of lateral anodes beneath the surface anode rod (6.6 ± 0.2 cm) that are coupled to the same voltage source. Design 2 of the SSAV-(LA-PCPSS) includes two pairs of lateral anodes beneath the surface anode rod (6.6 ± 0.2 cm and 13.4 ± 0.2 cm) m) that are coupled to the same voltage source. Design 3 of the DSAV-(LA-PCPSS) has a pair of lateral anodes beneath the surface anode rod (6.6 ± 0.2 cm) that are coupled to distinct voltage sources. Design 4 of the DSAV-(LA-PCPSS) incorporates two pairs of lateral anodes beneath the surface anode rod (6.6 ± 0.2 cm and 13.4 ± 0.2 cm) that are coupled to distinct voltage sources.

Under the effect of an electric field, Ni was removed from the surface layers and accumulated in the subsequent layers (middle of the apparatus) using the ideal/traditional design of the PCPSS (Fig. 9).

Small amounts of Ni were removed from the bottom layers compared to the surface layers. Design 1 of the SSAV-(LA-PCPSS) negatively affected the removed Ni, resulting in migration towards the anode surface. Design 2 of the SSAV-(LA-PCPSS) improved the removal of Ni adjacent to the lateral anodes. The removal of Ni was improved with applying design 3 of the DSAV-(LA-PCPSS) compared to the ideal/traditional design of the PCPSS and designs 1 and 2 of the SSAV-(LA-PCPSS). This improvement was enhanced in the surface layers, while Ni accumulation was observed close to the cathode rods (located between cathode pipes) when design 4 of the DSAV-(LA-PCPSS) was used. As a consequence, design 3 of the DSAV-(LA-PCPSS) provided the best remediation outcomes for Ni-containing real contaminated soil. The removal of Zn was comparable to that of Ni, although no Zn accumulation was seen around the cathode rods in design 4 of the DSAV-(LA-PCPSS) (Fig. S7). As a result, design 4 of the DSAV-(LA-PCPSS) is regarded as the optimum option for Zn removal, ensuring optimal remediation from either the surface or bottom layers.

Figure S8 depicts the behavior of Pb in real polluted soil under the effect of several SEKR-PCPSS systems. In the ideal/traditional PCPSS design, Pb was removed from the top layer and re-accumulated in the following layers. Applying design 1 of the SSAV-(LA-PCPSS) increased the removed Pb from the area next to the lateral anodes, while reverse migration of Pb towards the surface anode was observed. Installing more lateral anodes in design 2 of the SSAV-(LA-PCPSS) enhanced the removed Pb adjacent to the lateral anodes when compared to the ideal/traditional design of the PCPSS and design 1 of the SSAV-(LA-PCPSS). Pb reverse migration towards the anode surface was also found in design 2 of the SSAV-(LA-PCPSS). Pb removal was improved with designs 3 and 4 of the DSAV-(LA-PCPSS), which were identical. As a result, designs 3 and 4 of the DSAV-(LA-PCPSS) are deemed suitable for the removal of Pb from actual contaminated soils.

Figure S9 depicts the remediation of Cd-contaminated real soils utilizing multiple designs, including the PCPSS, the SSAV-(LA-PCPSS), and the DSAV-(LA-PCPSS). In the ideal design of the PCPSS, the Cd was removed from the surface layers and re-accumulated in the subsequence layer. The removal of Cd from the surface later was better than that observed in the bottom layers. Applying designs 1 and 2 of the SSAV-(LA-PCPSS) had a detrimental impact on the regular removal accomplished by the ideal/traditional design of the PCPSS, and reverse migration of Cd towards the surface anode rod was observed. Using DSAV-(LA-PCPSS) designs 3 and 4 resulted in an increase in the amount of Cd removal. Design 4 of the DSAV (LA-PCPSS) removed more Cd than design 3. As a result, design 4 of the DSAV-(LA-PCPSS) deemed appropriate for the remediation of Cd-contaminated real soil. The behavior of Cr in the presence of electric field utilizing various PCPSS designs, including the SSAV-(LA-PCPSS) and the DSAV-(LA-PCPSS), is depicted in Fig. S10. It was observed the irregular removal of Cr inside the SEKR apparatuses. In design 1, the removed Cr was amplified in the area close to the lateral anodes, while design 2’s second pair of lateral anodes showed comparable behavior. The best removal of Cr contaminated soil from the area around the surface anode rod was observed in the following order: design 4 of the DSAV-(LA-PCPSS) > design 3 of the DSAV-(LA-PCPSS) > the ideal/traditional design of the PCPSS > design 1 of the SSAV-(LA-PCPSS) > design 2 of the SSAV-(LA-PCPSS). As a result, design 4 of DSAV-(LA-PCPSS) is regarded as the best option for Cr removal.

Design 1 of the SSAV-(LA-PCPSS) has a pair of lateral anodes beneath the surface anode rod (6.6 ± 0.2 cm) that are coupled to the same voltage source. Design 2 of the SSAV-(LA-PCPSS) includes two pairs of lateral anodes beneath the surface anode rod (6.6 ± 0.2 cm and 13.4 ± 0.2 cm) m) that are coupled to the same voltage source. Design 3 of the DSAV-(LA-PCPSS) has a pair of lateral anodes beneath the surface anode rod (6.6 ± 0.2 cm) that are coupled to distinct voltage sources. Design 4 of the DSAV-(LA-PCPSS) incorporates two pairs of lateral anodes beneath the surface anode rod (6.6 ± 0.2 cm and 13.4 ± 0.2 cm) that are coupled to distinct voltage sources.

Cu was removed from the surface layers using the ideal/traditional PCPSS design and re-accumulated in the subsequent layers, but applying designs 1 and 2 of the SSAV-(LA-PCPSS) has a detrimental effect on Cu migration towards cathodes pipes (Fig. S11). Designs 1 and 2 of the SSAV-(LA-PCPSS) showed increased Cu removal in the area next to the lateral anodes. Using designs 3 and 4 of the DSAV-(LA-PCPSS) enhanced the amount of Cu removal. Design 3 of the DSAV-(LA-PCPSS) increased Cu depletion in the area close to the cathode rod compared with design 4. Design 4 of the DSAV-(LA-PCPSS) reduced the accumulation of Cu in the apparatus middle compared to design 3. As a result, designs 3 and 4 of DSAV-(LA-PCPSS) are judged suitable for Cu removal. The ideal/traditional design of the PCPSS efficiently removed Sr from the surface layers, however, applying designs 1 and 2 of the SSAV-(LA-PCPSS) had a negative effect on this removal (Fig. S12). Applying DSAV-(LA-PCPSS) designs 3 and 4 resulted in higher Sr removal percentages than the optimal PCPSS design and SSAV-(LA-PCPSS) designs 1 and 2. Design 4 of the DSAV-(LA-PCPSS) provided the best results for Sr removal when compared to design 3. As a result, design 4 of the DSAV-(LA-PCPSS) is deemed the most suited design for the removed Sr. Fig. S13 depicts the distribution of Mn in response to various electric field designs. The ideal/traditional PCPSS design, designs 3 and 4 of the DSAV-(LA-PCPSS), produced the best Mn removal rats from the surface layers. Using DSAV-(LA-PCPSS) designs 3 and 4 increased the amount of Mn removed from the bottom layers compared with the ideal/traditional PCPSS design. Applying design 1 of the SSAV-(LA-PCPSS) did not result in a substantial increase in the removed Mn compared to the ideal/traditional PCPSS design, however more Mn was accumulated in the middle of the apparatus. Applying the design 2 of the SSAV-(LA-PCPSS) had a negative impact on remediation efficiency and resulted in a lower removal rate than the other designs. Thus, designs 3 and 4 of the DSAV-(LA-PCPSS) are suitable for SEKR of Mn-contaminated soil.

#### Evaluation of EO flow and current passing during confirmation experiments

**Fig. 10:**
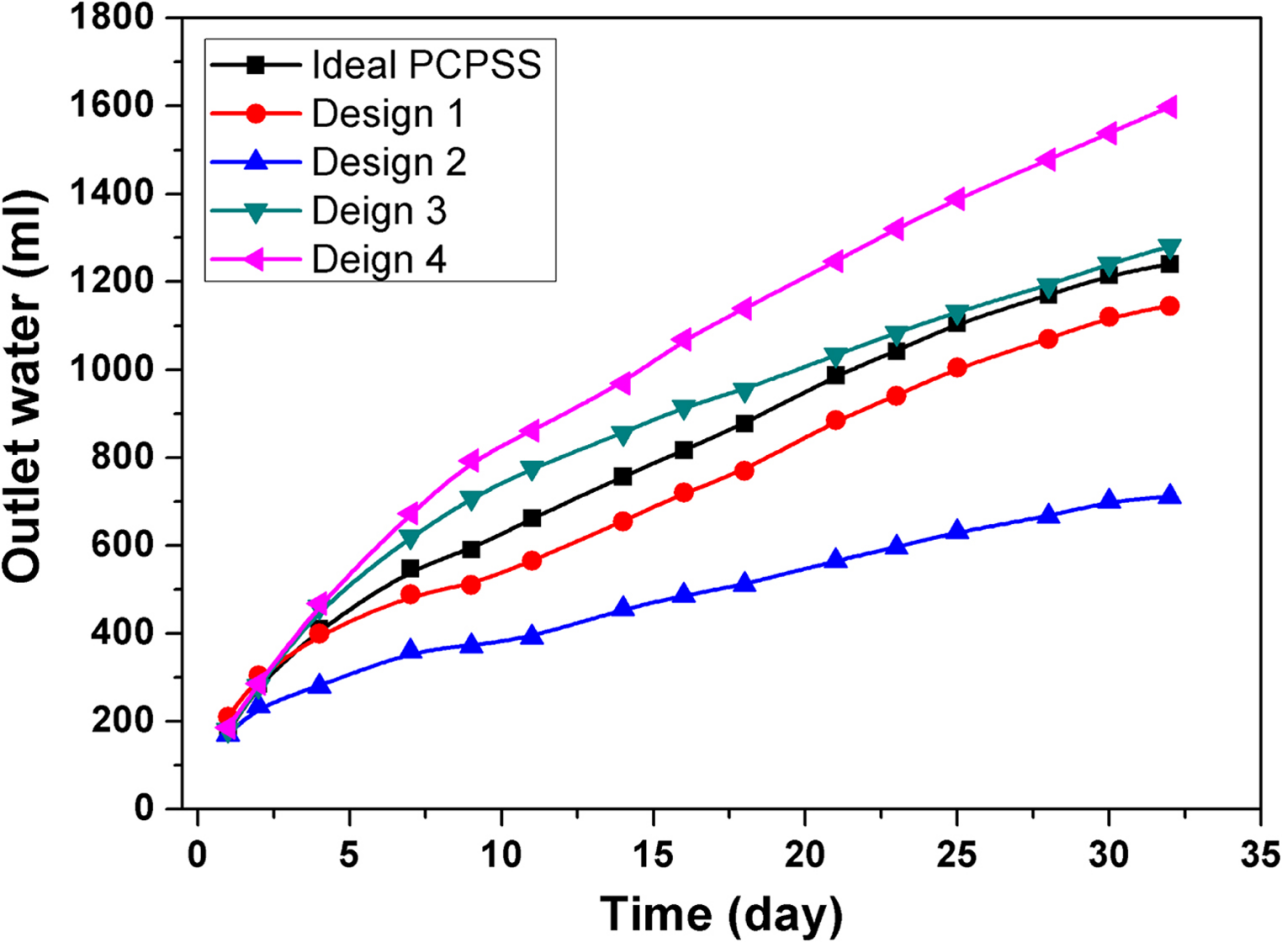
The effect of SEKR-PCPSS different designs on the EO rate/dewatering.

Figure 10 shows the influence of several PCPSS designs on the EO rate/dewatering for confirmation studies using areal-contaminated soil. The EO rate/dewatering for the five designs was listed in descending order as follows: design 4 of the DSAV-(LA-PCPSS) > design 3 of the DSAV-(LA-PCPSS) > the ideal/traditional design of the PCPSS > design 1 of the SSAV-(LA-PCPSS) > design 2 of the SSAV-(LA-PCPSS).

**Fig. 11:**
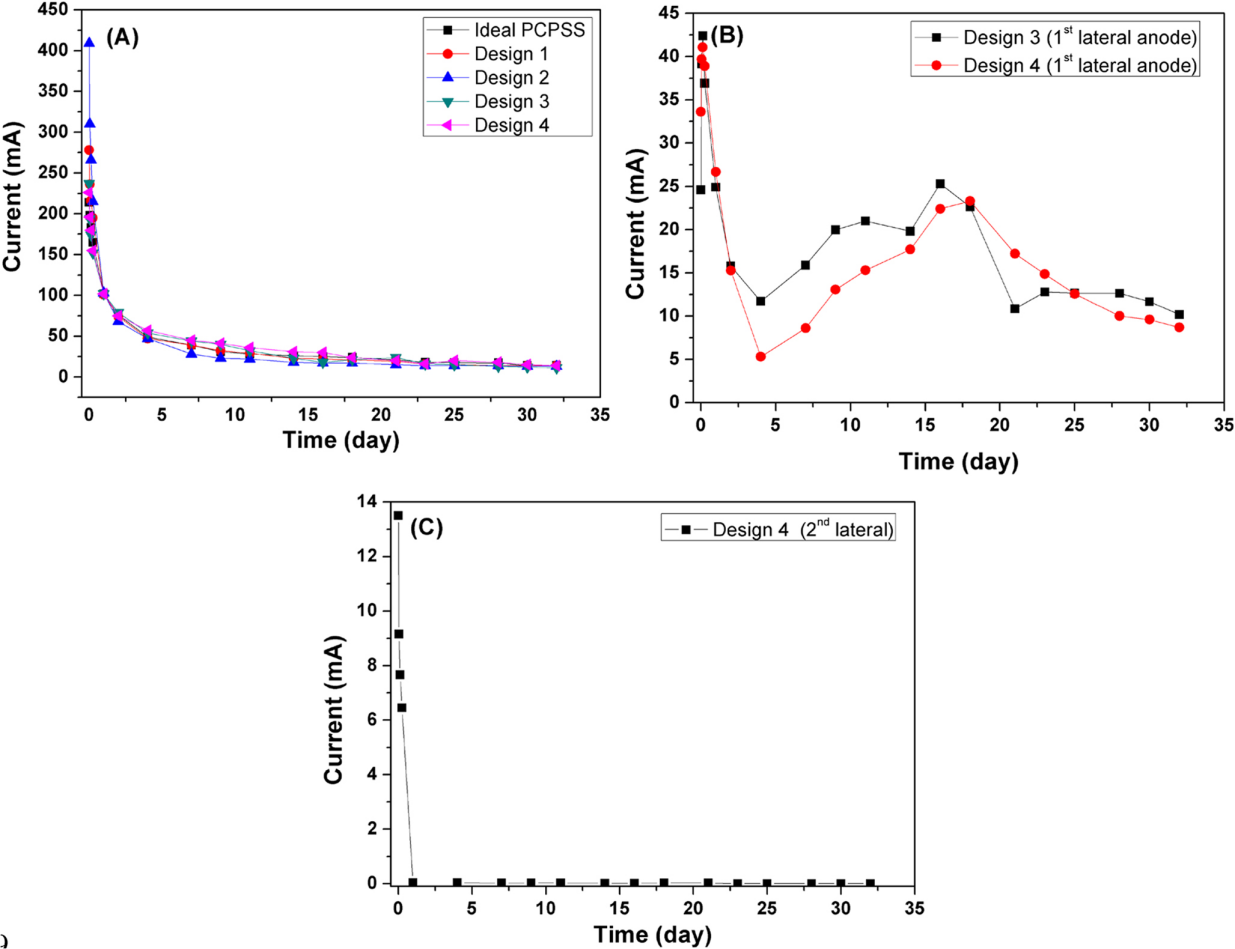
(**a**) Current passing from surface anode rod to bottom cathode pipes, (**b**) current passing from 1^st^ lateral anodes (beneath the surface anode, 6.6 ± 0.2 cm) to cathode graphite rod for designs 3 and 4, and (**c**) current passing from 2^nd^ lateral anodes (beneath the surface anode rod, 13.4 ± 0.2 cm) to cathode graphite rod for design 4.

The current passing values from the surface anode/anodes to the downward cathodes were similar for the various proposed designs of the PCPSS, the SSAV-(LA-PCPSS), and the DSAV-(LA-PCPSS), as depicted in Fig. 11a. Figure 11b depicts the current passing from the first pair of lateral anodes for designs 3 and 4 of the DSAV-(LA-PCPSS). Design 3 had higher current passing values than design 4 for 3–15 days, but decreased to around 10 mA by the end of the trial. After 24 h, the current going through the second pair of lateral anodes of design 4 of the DSAV-(LA-PCPSS) was extremely low (Fig. 11c). The DSAV-(LA-PCPSS) considerably did not consume more energy than the ideal/traditional PCPSS as a result of the installation of lateral anodes; this is considered an economic benefit for the SEKR.

Design 1 of the SSAV-(LA-PCPSS) has a pair of lateral anodes beneath the surface anode rod (6.6 ± 0.2 cm) that are coupled to the same voltage source. Design 2 of the SSAV-(LA-PCPSS) includes two pairs of lateral anodes beneath the surface anode rod (6.6 ± 0.2 cm and 13.4 ± 0.2 cm) m) that are coupled to the same voltage source. Design 3 of the DSAV-(LA-PCPSS) has a pair of lateral anodes beneath the surface anode rod (6.6 ± 0.2 cm) that are coupled to distinct voltage sources. Design 4 of the DSAV-(LA-PCPSS) incorporates two pairs of lateral anodes beneath the surface anode rod (6.6 ± 0.2 cm and 13.4 ± 0.2 cm) that are coupled to distinct voltage sources.

## Conclusions

The present work improved the PCPSS by installing lateral anodes utilizing two approaches: (a) the SSAV-(LA-PCPSS) and (b) the DSAV-(LA-PCPSS). The experimental results revealed that based on the signal-to-noise ratio released from the Taguchi approach (L_9_OA), the DSAV-(LA-PCPSS) could be optimized at an applied voltage of 1 V cm^−1^ for the surface and the first and second lateral anodes. After conducting basic investigations utilizing the Taguchi approach to optimize the performance of DSAV-(LA-PCPSS), indigenous Sr demonstrated the best remediation response compared to Ni and other indigenous elements (Zn, Pb, Cr, Cu, and Mn). According to the Taguchi analysis, installing lateral anodes (position B) increased the EO/dewatering rate when linked to a low applied voltage (0.5 V cm^−1^). Applying the SSAV-(LA-PCPSS) resulted in the reverse migration of some inorganic pollutants. In comparison to the ideal/traditional designs of the PCPSS and the SSAV-(LA-PCPSS), the DSAV-(LA-PCPSS) is thought to be an appropriate design for the SEKR of inorganic pollutants. Design 3 of the DSAV-(LA-PCPSS) is more appropriate for SEKR Ni, while, design 4 is more appropriate for Zn, Cd, Cr, and Sr. However, designs 3 and 4 are more suitable for SEKR of Pb and Cu. The DSAV-(LA-PCPSS) application increased the dewatering/EO flow rate with comparatively little additional energy use, which is considered an economic benefit for the SEKR study.

## Supplementary Information

Supplementary Material 1.

Supplementary Material 2.

Supplementary Material 3.

Supplementary Material 4.

Supplementary Material 5.

Supplementary Material 6.

Supplementary Material 7.

Supplementary Material 8.

Supplementary Material 9.

Supplementary Material 10.

Supplementary Material 11.

Supplementary Material 12.

Supplementary Material 13.

## Data Availability

The datasets used and/or analysed during the current study available from the corresponding author on reasonable request.

## Abbreviations

SEK: Soil electrokinetics
SEKR: Soil electrokinetics remediation
PCPSS: Perforated cathode pipe SEKR system
LA-PCPSS: Lateral anodes- perforated cathode pipe SEKR system
DSAV: Different sources of applied voltages
SSAV: The same source of applied voltage
EO: Electroosmosis
DC: Direct current
S/N: The signal-to-noise ratio
CR: Concentration ratio
Indigenous: Elements found in the tested soil without artificial pollution
