# Supplementary material for: Optimizing electrokinetic remediation for pollutant removal and electroosmosis/dewatering using lateral anode configurations
Source: Sci Rep. 2024 Oct 25;14:25380. doi: 10.1038/s41598-024-75060-6 (PMC11512046; doi:10.1038/s41598-024-75060-6)
Supplement: Supplementary file 4 — Supplementary Material 4. [file 41598_2024_75060_MOESM4_ESM.docx]

**Fig. S4**

Fig. S4. Distribution of indigenous Cr within the DSAV-(LA-PCPSS) over the nine trials proposed by the Taguchi approach.


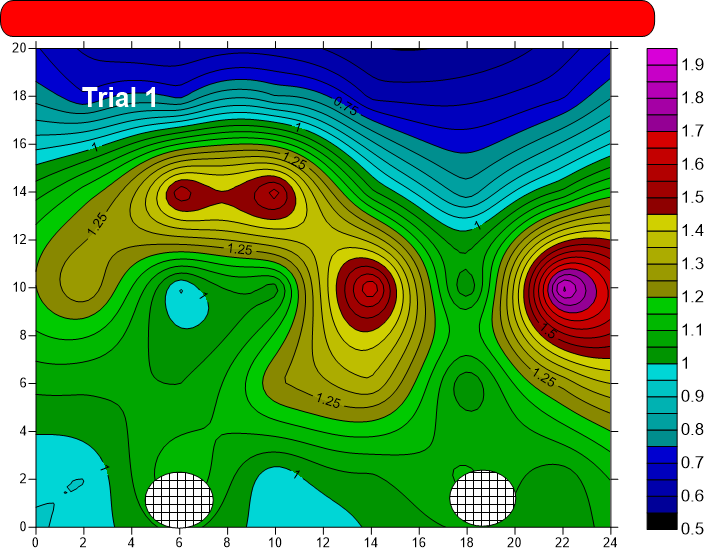

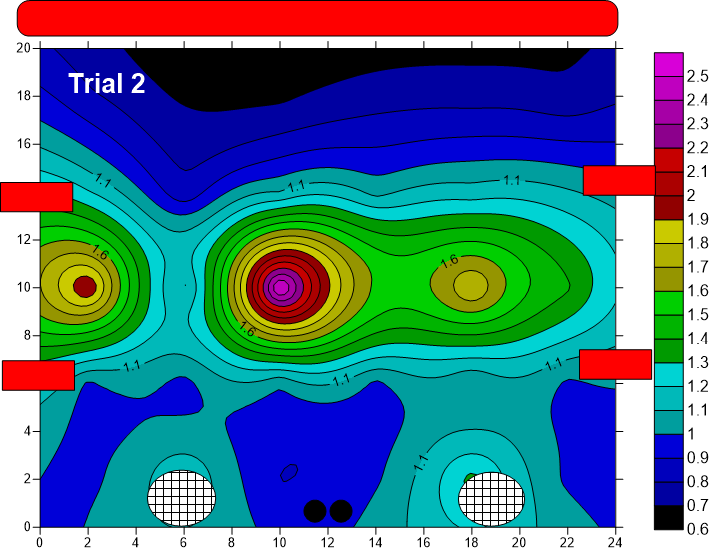

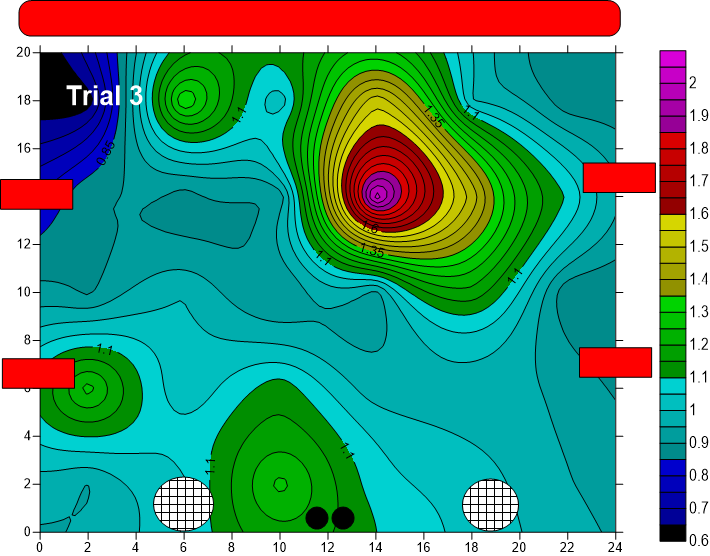

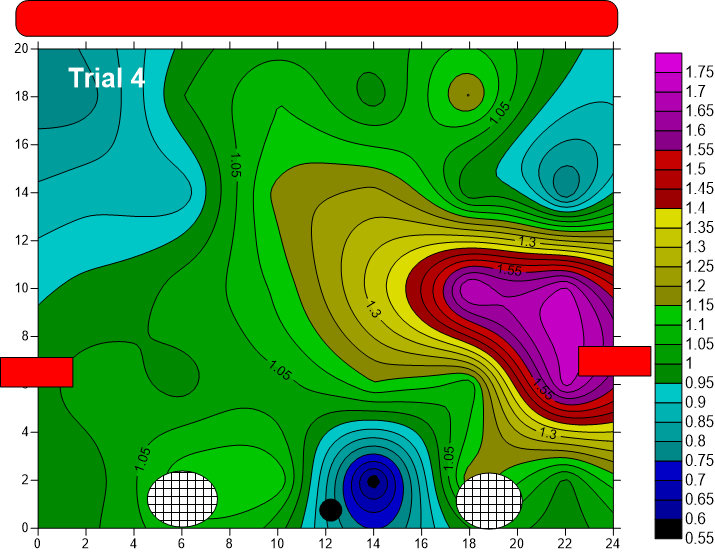

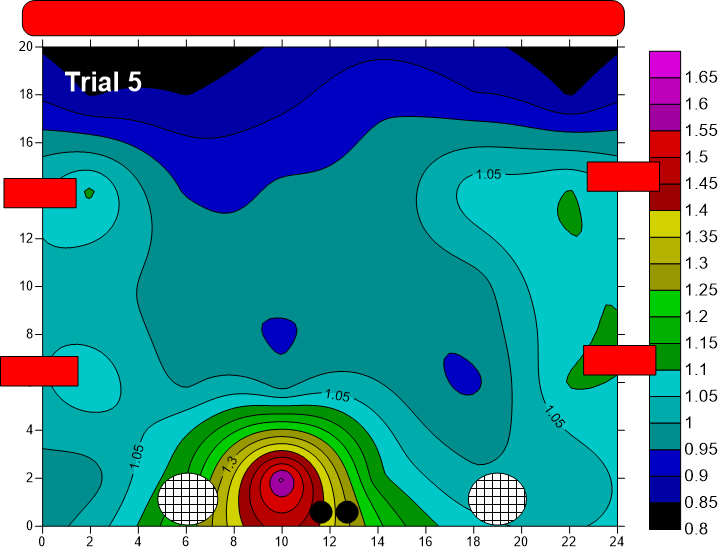

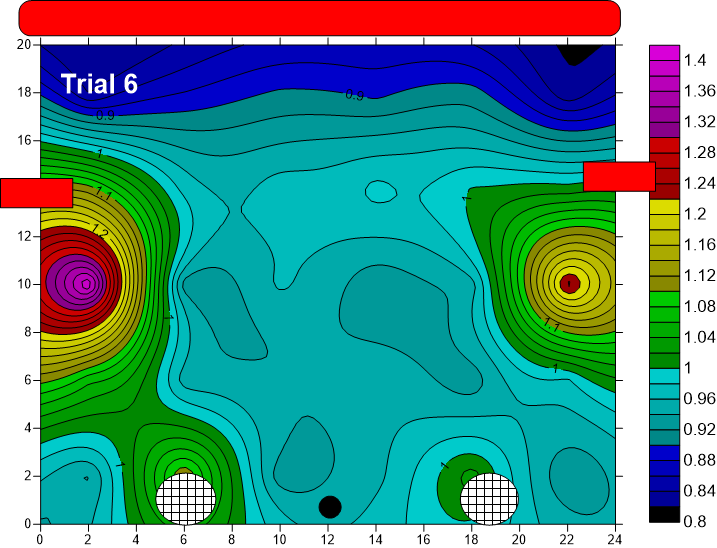

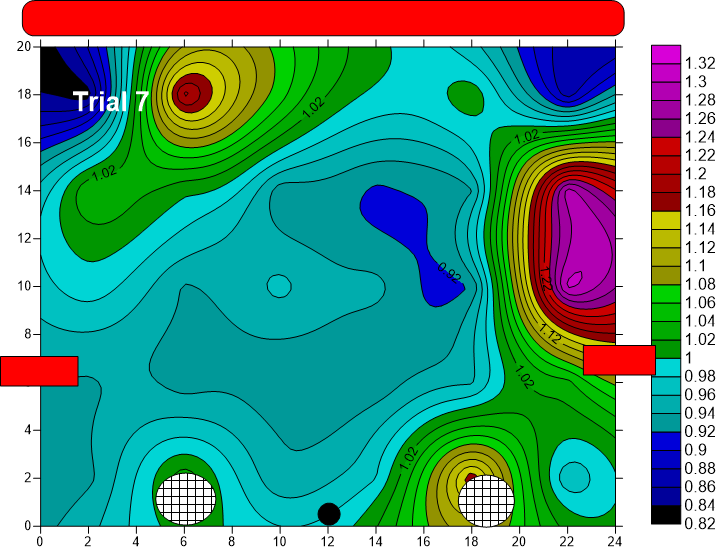

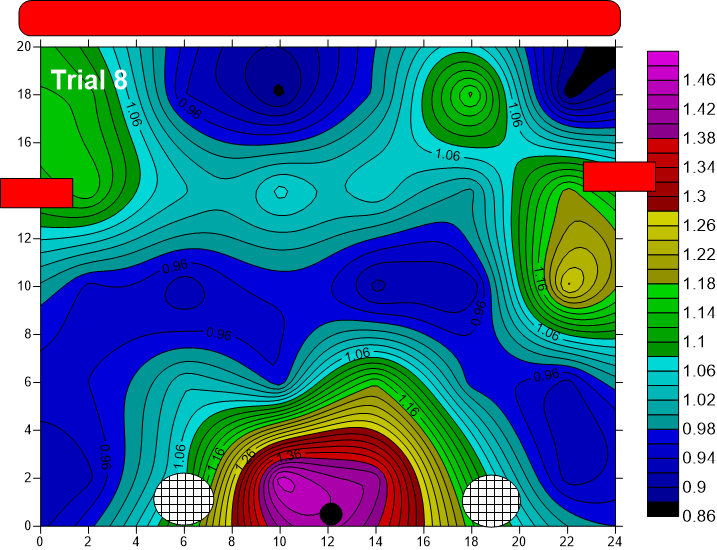

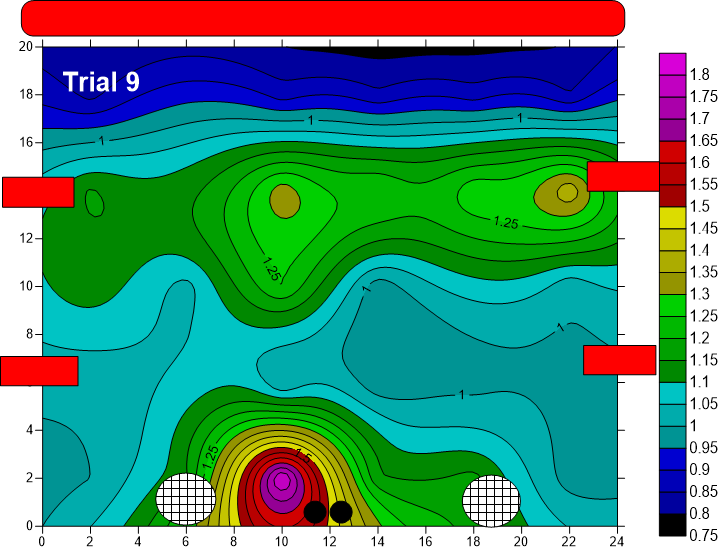


**Cathode pipes**

**Anode**

**Cathodes nails**

**Lateral anodes**
