# Supplementary material for: Optimizing electrokinetic remediation for pollutant removal and electroosmosis/dewatering using lateral anode configurations
Source: Sci Rep. 2024 Oct 25;14:25380. doi: 10.1038/s41598-024-75060-6 (PMC11512046; doi:10.1038/s41598-024-75060-6)
Supplement: Supplementary file 9 — Supplementary Material 9. [file 41598_2024_75060_MOESM9_ESM.docx]

Fig. S9


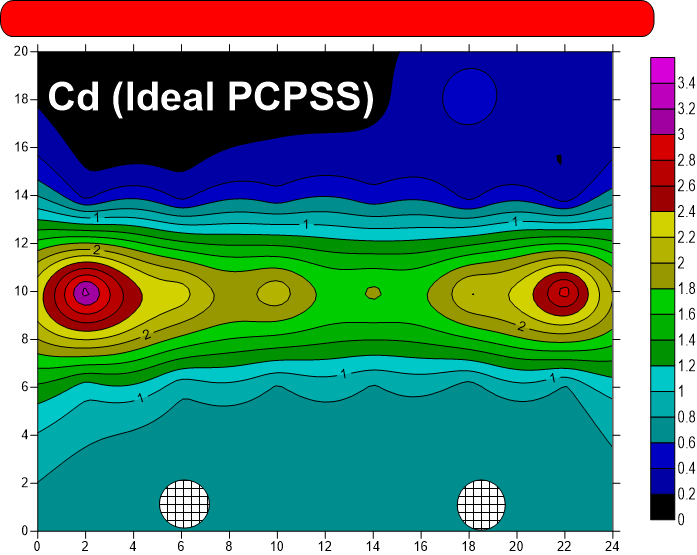

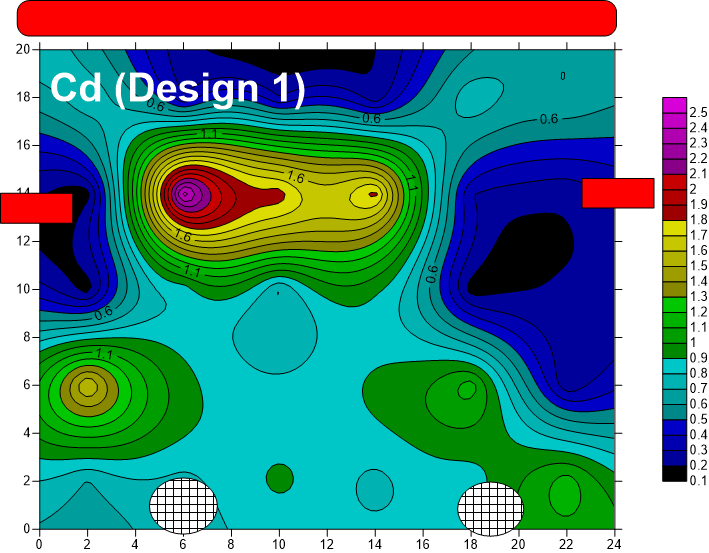

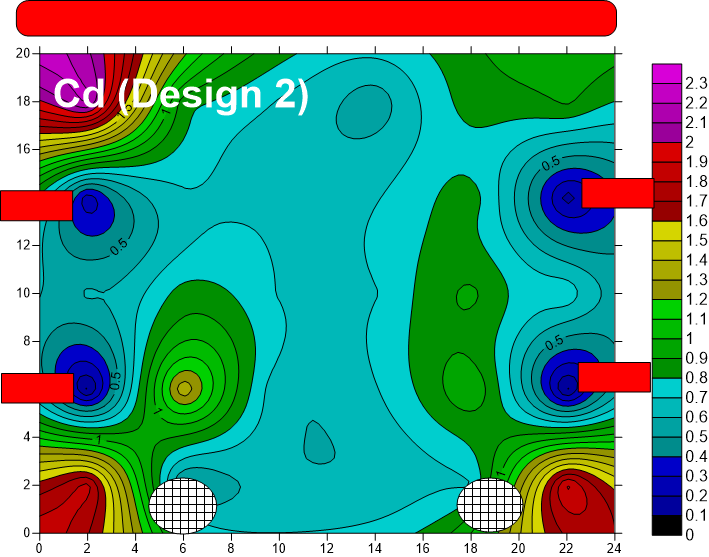

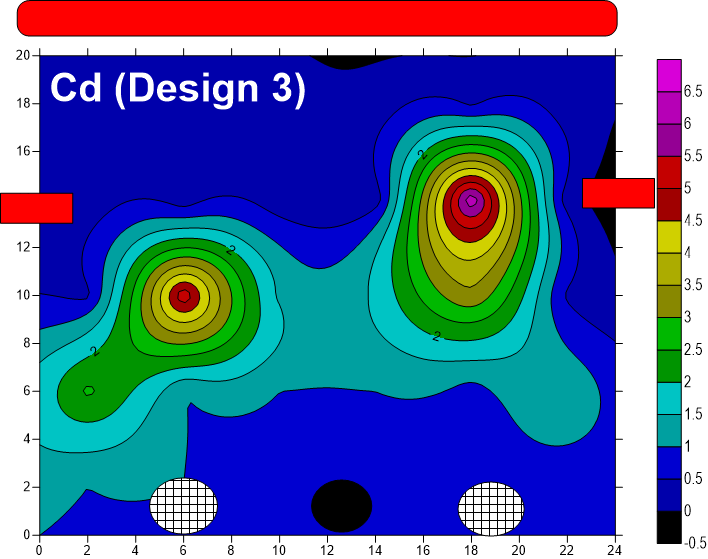

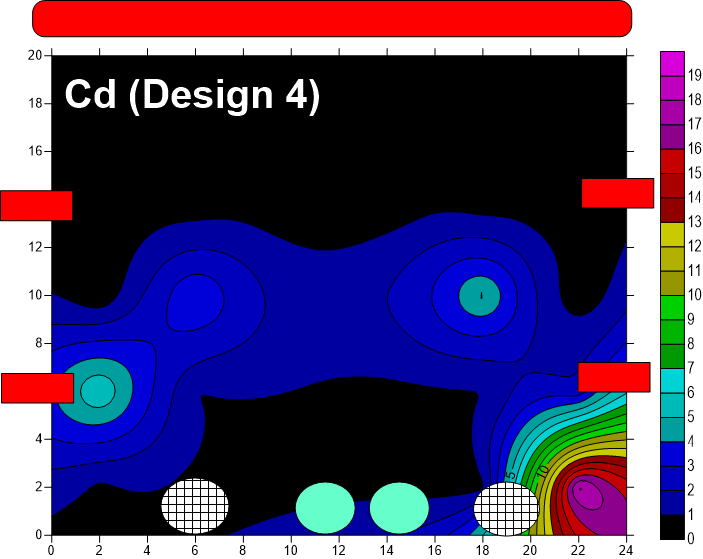


Fig. S9. Removal of Cd from real contaminated soil using the ideal and improved designs of the PCPSS. Design 1 of the SSAV-(LA-PCPSS) has a pair of lateral anodes beneath the surface anode rod (6.6±0.2 cm) that are coupled to the same voltage source. Design 2 of the SSAV-(LA-PCPSS) includes two pairs of lateral anodes beneath the surface anode rod (6.6±0.2 cm and 13.4±0.2 cm) m) that are coupled to the same voltage source. Design 3 of the DSAV-(LA-PCPSS) has a pair of lateral anodes beneath the surface anode rod (6.6±0.2 cm) that are coupled to distinct voltage sources. Design 4 of the DSAV-(LA-PCPSS) incorporates two pairs of lateral anodes beneath the surface anode rod (6.6±0.2 cm and 13.4±0.2 cm) that are coupled to distinct voltage sources.
